# Host plant selects bacterial rhizosphere microbiome function whereas community structure is determined by soil legacy

**DOI:** 10.1093/ismeco/ycag083

**Published:** 2026-03-28

**Authors:** Rodrigo G Taketani, Ian M Clark, Payton T O Yau, Li Liu, Fangbo Zhang, Gye-Ryeong Bak, Catriona M A Thompson, J Miguel Bonnin, Helen Stewart, Jacob G Malone, Susan Jones, Nicola Holden, Mathew J Ryan, Timothy H Mauchline

**Affiliations:** Rothamsted Research, West Common, Harpenden, Hertfordshire, AL5 2JQ, United Kingdom; Rothamsted Research, West Common, Harpenden, Hertfordshire, AL5 2JQ, United Kingdom; Department of Rural Land Use, Scotland’s Rural College, Aberdeen, Aberdeenshire AB21 9YA, United Kingdom; Rothamsted Research, West Common, Harpenden, Hertfordshire, AL5 2JQ, United Kingdom; Rothamsted Research, West Common, Harpenden, Hertfordshire, AL5 2JQ, United Kingdom; Rothamsted Research, West Common, Harpenden, Hertfordshire, AL5 2JQ, United Kingdom; John Innes Centre, Norwich Research Park, Colney Ln, Norwich, Norfolk NR4 7UH, United Kingdom; Centre of Agriculture and Biosciences International, CABI, Silwood Park, Buckhurst Road, Ascot, Berkshire, SL5 7PY, United Kingdom; Centre of Agriculture and Biosciences International, CABI, Silwood Park, Buckhurst Road, Ascot, Berkshire, SL5 7PY, United Kingdom; John Innes Centre, Norwich Research Park, Colney Ln, Norwich, Norfolk NR4 7UH, United Kingdom; School of Biological Sciences, University of East Anglia, Norwich, Norfolk NR4 7TJ, United Kingdom; Information and Computational Sciences, The James Hutton Institute, Invergowrie, Dundee, Angus, DD2 5DA, United Kingdom; Department of Rural Land Use, Scotland’s Rural College, Aberdeen, Aberdeenshire AB21 9YA, United Kingdom; Centre of Agriculture and Biosciences International, CABI, Silwood Park, Buckhurst Road, Ascot, Berkshire, SL5 7PY, United Kingdom; Rothamsted Research, West Common, Harpenden, Hertfordshire, AL5 2JQ, United Kingdom

**Keywords:** rhizosphere microbiome, soil legacy, host plant selection, microbial functional traits, crop-specific microbiome assembly

## Abstract

The drivers between host plant, associated rhizosphere microbiome functions, and related plant health implications are complex and a field of continuous development. Furthermore, understanding of the interplay between soil, plant, and microbiome across different plant species and contrasting geographical areas is scarce. The United Kingdom (UK) Crop Microbiome Cryobank project, the world’s first open crop/soil microbiome resource can fill this research gap. It utilizes contrasting UK soil types, with comprehensive environmental and agronomic metadata and has generated associated rhizosphere and bulk soil microbiome information for six crops (wheat, barley, oats, fava beans, oilseed rape, and sugar-beet) including a bacterial culture collection and 16S rRNA gene datasets. Here, using functional and taxonomic data from 24 000 bacterial cultures and 315 16S rRNA gene metabarcoded soil libraries, we show that geographical location and soil environment primarily influence the phylogeny of rhizosphere bacterial communities, whereas crop genotype is key in determining the function of associated rhizosphere microbiota. Sugar-beet and oilseed rape predominantly select for drought tolerant microbes, barley for Zn-solubilizing microbes and fava bean has a reduced selection of N-mineralizing microbes. These findings emphasize the need to consider the host plant’s developmental requirements and edaphic factors for successful deployment of microbiome facilitated agriculture.

## Introduction

The widely accepted definition of a microbiome is the microbial community inhabiting a defined habitat and its theater of activity [1]. They are found in every habitable environment on Earth and their diversity varies with the characteristics of the environment [1]. Soil microbiota are essential components of agricultural ecosystems [2]. They carry out a wide range of key steps in nutrient cycling, including nitrogen fixation and phosphorous solubilization, improving soil structure through the production of extracellular polymers and filamentous growth, pathogen suppression, plant growth promotion (PGP) through the production or breakdown of phytohormones [3] and improved plant abiotic stress tolerance [4].

Since the late 1800s, agricultural practices have leveraged the use of soil microorganisms to improve crop productivity and sustainability [5–7]. In 2020, this industry amounted to US$2.9 Bn globally with a broad variety of products for different crops and different PGP capabilities [8]. These products are derived from a variety of taxonomic groups such as *Rhizobium*, *Azotobacter*, *Bacillus*, and *Trichoderma*, and are applied to a wide range of crop species [9].

Amendment of agricultural systems with plant growth promoting microbes has had mixed success [10]. Thus, understanding how microbiomes interact with their environment and host is key to the development of better inoculants and agricultural practices that leverage the soil microbiome. The foundation of the UK Crop Microbiome Cryobank (UKCMCB) project is the utilization of contrasting soil types from across the UK, with strong environmental and agronomic metadata, for controlled environment growth of six important crops [i.e. spring wheat (*Triticum aestivum*), spring barley (*Hordeum vulgare*), spring oats (*Avena sativa*), fava beans (*Vicia faba*), oilseed rape (*Brassica napus*), sugar beet (*Beta vulgaris* subsp. Vulgaris)] along with associated bulk soils [11].

This design was used to generate a sample bank of tens of thousands of bacterial cultures, aligned with soil and cropping history metadata, which were characterized for PGP functions. In addition, partial 16S rRNA gene information of pooled isolates for each sample was obtained. Furthermore, over 300 rhizosphere/bulk soil deoxyribonucleic acid (DNA) samples were subjected to culture independent 16S rRNA gene amplicon sequencing [12]. The multifaceted design of the project provides an invaluable resource to investigate soil–crop–microbiome interactions.

Plant–microbe relationships are known to be mediated by a series of complex chemical signals between host and microbes [13] and these communication systems are represented across the microbial tree-of-life [13]. In the same manner, PGP functions are also dispersed among a diverse cohort of microbes [9], generating a level of functional redundancy in the community that is believed to support a level of resilience in the soil system [14]. Nitrogen fixation, for example, is an important step in the nitrogen cycle that has a major impact on soils, with bacterial families such as Rhizobiaceae, Frankiaceae, and Burkholderiaceae typically fixing nitrogen in soils in association with their host plant [15]. Conversely, the intra-specific diversity created by genome plasticity and mobile genetic elements allows strains from the same species to occupy different niches [16]. Thus, the high diversity of soil microbial communities and functional redundancy allow different facets of the microbiome to respond differently to environmental challenges. On a local scale, historical legacies, natural or by human interventions alters the soil and its associated microbial community, which in turn leads to different responses to current events [17]. This soil legacy is particularly important in agricultural soils where management can affect how nutrients are cycled and other ecological services [18, 19].

In this study, we test the hypothesis that crop–microbiome and soil–microbiome interactions differentially shape the root microbiome in terms of taxonomic and functional selection. With this aim, we leveraged the breadth and depth of data produced by the UKCMCB [11] to assess how different crops, soils, and geographic locations shape the root-associated soil bacterial community structure and PGP function at an unprecedented scale across UK arable farming systems. Our results indicate that, isolation site emerged as the primary driver of soil bacterial community taxonomic discrimination. However, the functional traits associated with soil microbes were primarily driven by the plant species grown in a given soil, regardless of soil type or geographic origin.

## Materials and methods

### Sample selection and glasshouse experiment

The sampling locations used in this study were derived from the ASSIST farm network (comprising 330 samples), a study farm network of commercial farms from across the UK (https://farmpep.net/project/assist), and supplemented with field sites from Rothamsted Research, the James Hutton Institute and a commercial field site via SRUC. They were selected according to their texture as described previously [11]. Briefly, soil texture characteristics (silt, clay, and sand percentages) were clustered using Complete Linkage and split into three different major soil groups. For each of these three groups, three locations with pH between 6 and 7 were selected. Soil chemical and physical characteristics can be found on the agMicroBiome Base website (https://agmicrobiomebase.org) and on the supplementary material spreadsheet. At the selected locations (Fig. S1), 300 kg of soil was sampled and transported to Rothamsted Research. Each soil was sieved through a 5 mm mesh, mixed thoroughly and used in the potting experiments.

In each of these soils, seeds were sown for crops of high economic importance to the UK using varieties in the 2021 AHDB recommended list (https://ahdb.org.uk). These crops and varieties were spring wheat (Mulika), spring oats (WPB Elyann), spring barley (RGT-Planet), fava beans (Lynx), oilseed rape (Campus), and sugar beet (Degas). Seeds were surface sterilized with 70% ethanol for 5 minutes and washed for 1 minute with sterile deionized water. Four seeds were sown in each pot and grown in a glasshouse until flowering stage, with a day/night cycle of 16/8 hours and temperatures of 20/18°C and watered every other day. For the cereals and beans 100 cm^2^ square pots were used, whereas for the other crops larger 20 cm diameter pots were used. After germination plants were thinned so that only one plant was maintained in each pot. For each soil type, control pots without plants were incubated along with the other pots. For each location (*n* = 9) and crop or bulk soil (*n* = 7) combination five replicate pots were split randomly across five replicate blocks.

When plants reached flowering (anthesis) stage, they were not watered for 48 hours before sampling, to facilitate the harvesting of rhizosphere soil. The soils potted without plants (bulk soils) were sampled after ten weeks discarding the top one centimeter and three centimeters from the edges of the pot. The crop rhizosphere samples were collected by removing root-containing soil from each pot and gently removing the bulk soil surrounding the roots into a sterile bag. The soil closely attached to the roots was then shaken into a new sterile bag to harvest the rhizosphere soil. Individual bulk and rhizosphere soil samples were homogenized by shaking and then divided into separate tubes for storage at −20°C for DNA extraction and at 4°C for bacterial culture isolations. For this study, each pot will be hereafter be referred to as a sample. DNA from bulk soil and rhizosphere samples were extracted as previously described [12].

### Culture independent 16S ribosomal ribonucleic acid gene metabarcoding

The uncultivated bacterial community dataset used in this study is part of AgMicroBiome Base. It can be found in European Nucleotide Archive (ENA) under accession (PRJEB58189). This data was presented in a previous study [12] and we are reanalysing it here to compare with the culture dependent approach. The data processing is described previously [12] (https://github.com/HuttonICS/agmicrobiomebase). In addition to the processing described previously ASVs that were present in less than 50% of the replicates were excluded. The data was normalized by rarefaction so that all libraries had the same number of sequences as the smallest using phyloseq [20]. Using the reference sequences file outputted by DADA2 [21] a phylogenetic tree was constructed using align-to-tree-mafft-fasttree from the QIIME2 package and added to the phyloseq object. The sample data at the https://agmicrobiomebase.org/data/ was also added to this object.

### High-throughput bacterial culture isolations

The soils from the potting experiment were serially diluted in sterile deionized water. Next, 0.1 ml of the soil solutions were plated onto 1/10^th^ Tryptic Soy Agar (TSA). The plates were incubated for 7 days at room temperature (~20°C). Plates containing between 30–300 colonies were used for the isolation of bacterial cultures. From each sample, 96 isolated colonies were randomly selected and transferred to 150 ml 1/10^th^ Tryptic Soy Broth (TSB) and grown in 96-well plates under the same conditions. These cultures were cryopreserved as described previously [11, 22], their PGP characteristics were tested, and their 16S rRNA genes/amplicons sequenced as described below. Since single colonies picked were not re-streaked to test for purity, from this point, we will refer to them as cultures instead of isolates or strains. Of these cultures, 24 192 were characterized for PGP functions and 16S rRNA gene sequenced in pools of 96 (representing an individual sample).

### Plant growth promoting high-throughput tests

Four of five replicated samples were randomly chosen to test the PGP potential of the obtained cultures, totaling 24 192 bacterial cultures. The bacterial cultures were tested for their potential to improve nutrient acquisition through phosphorus solubilization, potassium solubilization, casein hydrolysis, and siderophore production [23] (see Supplementary Methods for media details). Additionally, they were tested for functions associated with abiotic stress tolerance: ACC deaminase production, salt stress resistance, and drought tolerance [23] (see Supplementary Methods for media details). The bacterial cultures were transferred from the 96-well microtiter plates to square 12 cm Petri dishes using a 96-pin replicator for each respective medium. Cultures were grown for up to 14 days at room temperature in darkness. The assays were analysed qualitatively to minimize biases and between-plate variations; thus, the cultures were considered positive or negative for each test. The data containing per-culture results can be found at AgMicroBiome Base (https://agmicrobiomebase.org) and on the supplementary material, this table was used for all downstream analyses. Alongside these tests, the cultures were inoculated onto 1/10^th^ TSA to evaluate their recovery. A culture that was able to grow in at least one medium was considered recoverable. When calculating per-sample statistics, the number of cultures positive for a PGP function was normalized by dividing them by the number of recovered cultures.

### Cultured bacterial metabarcode sequencing

The DNA of the cultures obtained was extracted using a heat-shock protocol as follows. Firstly, for each culture, 5 ml of 4× lysis buffer (Tris 40 mM, EDTA 0.4 mM, 0.4% Triton-X) was mixed with 20 ml of 1/10th TSB culture (grown for 7 days at room temperature in darkness) mixed by pipetting. This solution was then incubated at 100°C for 15 minutes and cooled to 0°C on a thermocycler and then transferred to −20°C. After the cell lysis, 5 ml of each of the 96 lysates for each sample plate were mixed in a single tube and sent for sequencing. The 16S rRNA gene sequencing used primers 515F (GTGCCAGCMGCCGCG GTAA) and 907R (CCGTCAATTCCTTTGAGTTT) and was performed by Novogene using 250 bp pair-end sequencing kit in an Illumina® Novaseq 6000 sequencer according to the provider’s protocol. Sequences are available at ENA under accession (PRJEB58189).

The paired and trimmed sequences were processed using QIIME2 v.2024.05 [24] using Silva v138 16S rRNA [25] references for taxonomy assignment. The sequences were denoised and clustered using DADA2 [26]. Singletons were removed using the filter-table command. The phylogenetic tree was constructed using the align-to-tree-mafft-fasttree. The amplified sequence variants (ASV) taxonomic classification was performed using the classify-consensus-vsearch command using Silva v.138 references [25]. The files produced by QIIME2 were imported into R (https://cran.r-project.org) as a phyloseq [20] object. To eliminate possible contamination, ASVs with relative abundance below 0.05% were excluded. ASVs obtained from bacterial culture samples were named using the prefix OTU to differentiate those obtained from the uncultivated organisms that were named using the prefix ASV.

QIIME2 16S rRNA gene microbiome data (i.e. feature table, taxonomy, and phylogenetic tree) were imported to R as a phyloseq object. The sample data obtained from the AgMicrobiomeBase (https://agmicrobiomebase.org) was added to this object as sample metadata. This object was used for all downstream analysis.

### Data analysis and statistics

All data was analysed using R 4.2.1 in RStudio 2024.12.0 + 467. The alpha-diversity indexes were obtained using the picante 1.8.2 and vegan 2.6–8 packages [27]. The Shapiro–Wilk test was used to attest to the sample normality. When normality was rejected, we used Kruskal–Wallis to compare the groups and pairwise tests were done using Wilcoxon on rank-sum tests using Benjamin–Hochberg *P*-value adjustment. Factors that followed a normal distribution were evaluated using ANOVA followed by Tukey’s *post hoc* test. The NMDS analysis was performed using phyloseq. PERMANOVA, betadisper, and ANOSIM were performed using vegan 2.6–8 and pairwise PERMANOVA using the pairwiseAdonis package 0.4.1. Mantel tests were carried out using Spearman rho based on Bray–Curtis similarity matrixes using the vegan 2.6–8 package [27]. To account for the compositional nature of the sequencing data, we performed center log-ratio (clr) normalization of the data using *microbiome* package (REF) [28]. the data was used for principal component analysis (PCA) followed by Uniform Manifold Approximation and Projection for Dimension Reduction (UMAP) ordination [29] using uwot package [30]. The clr transformed ASV table was used for ANOSIM analysis using Aitchison distance. With compositionality and other technical biases in mind, the α-diversity was also estimated using the DivNet R package. [31]. The total community was constructed using a genus-level taxa table, while the isolates table was analysed using a class-level table. The latter level was used because it was the lowest level with a single taxon that belonged to all samples. It is important to highlight that we did not perform statistical analysis on our microbiome data with soil chemical or physical data because the latter was measured on soils that were collected on the farms and not from the potting experiment. The soil incubation time in the glasshouse will likely impact some soil parameters, leading to false associations between functions, taxa, or communities. All codes used in this paper can be found in Zenodo [32] (doi:10.5281/zenodo.19069134) and GitHub (https://github.com/rgtaketani/AgMicrobiome_paper) and metadata can be found in the supplementary material spreadsheet.

## Results

### Culture independent bacterial community α-diversity

**Figure 1:**
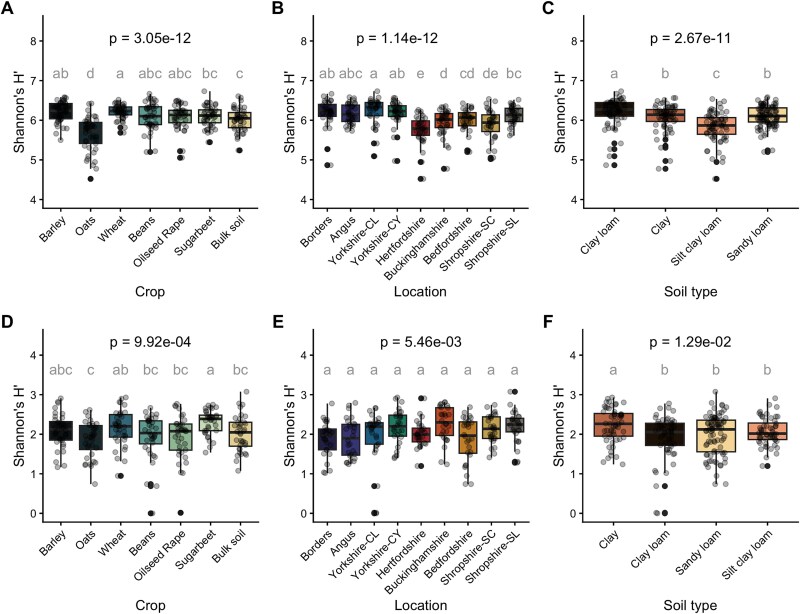
Shannon’s H′ alpha-diversity metrics based on 16S rRNA gene amplicon sequence variants (ASVs). Panels A–C show the culture independent bacterial community profiles, D–F the cultured bacterial profiles. A and D—Diversity index grouped on crop type, *n* = 45 and 36, respectively. B and E—Diversity index grouped on locations, *n* = 35 and 28, respectively. C and F—Diversity index based on soil texture type, *n* = 70 and 56, respectively (except for sandy loam *n* = 105 and 84, respectively). Boxplots show the distribution of diversity indexes across groups, with the central line representing the median, the box indicating the interquartile range (IQR), and whiskers extending to 1.5 × IQR. Each dot represents the diversity index for a sample. Treatments were compared with Kruskal–Wallis test followed by pairwise Wilcoxon rank sum exact test with Benjamin–Hochberg *P*-value adjustment. Letters above boxes represent the groups formed by Wilcoxon test.

The normalized features (ASVs) table was used for alpha-diversity metrics calculations (Fig. 1A–C, Fig. S2). The diversity indices show a significant variation between groups within the different factors (locations, crops, and soil types) based on Kruskal–Wallis (*P* < .001). The northernmost locations (Borders, Angus, Yorkshire-CL and CY) showed higher values based on the three indices (*n =* 35) (Fig. 1B, Fig. S2b, e, and  f), and the Hertfordshire sample had significantly lower values. When considering the different crops, oats had significantly lower diversity values than the other treatments for both diversity and richness (*n =* 45) (Fig. 1A and Fig. S2). In the case of soil type, clay loam had the highest values and silty clay loam the lowest species diversity and richness (Fig. 1C, Fig. S2c, f, and  i). We also observed a similar pattern for the Faith’s phylogenetic diversity (PD) and DivNet estimated Shannon’s H′ (for more details see Supplementary Results, Figs S7 and S8).

### Culture independent bacterial community β-diversity

**Figure 2:**
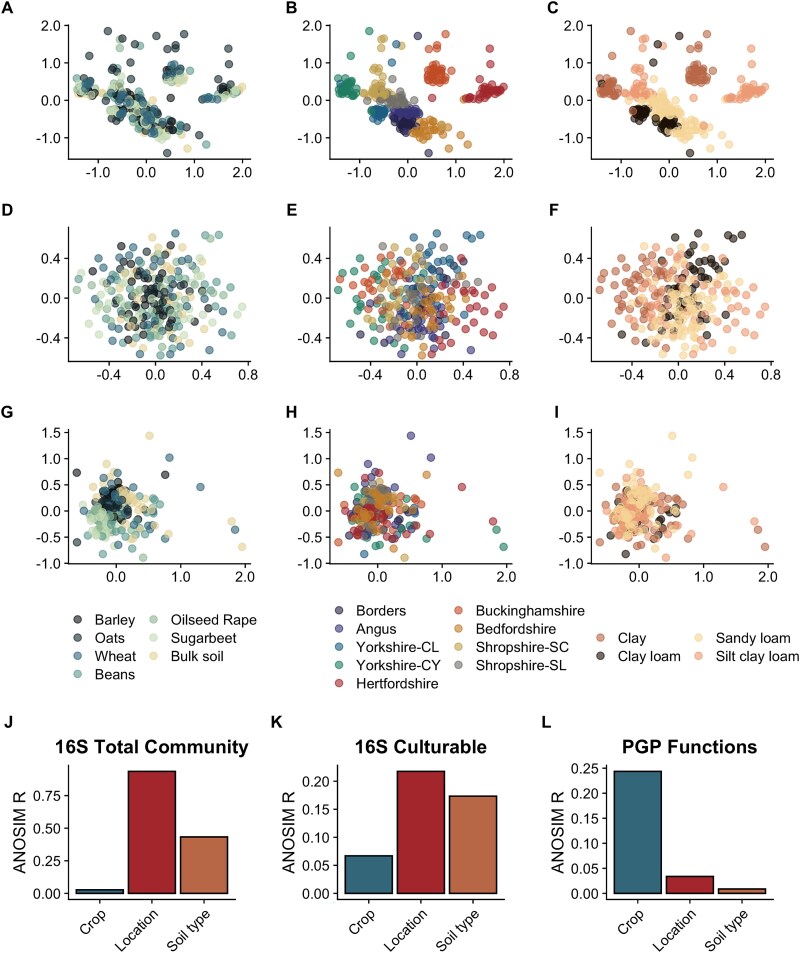
NMDS ordination of bacterial communities based on 16S rRNA gene ASVs and culture functional profiles and ANOSIM R values. NMDS plots based on Bray–Curtis dissimilarity. NMDS1 and NMDS2 are represented in the *x* and *y* axis, respectively. Panels A–C show the culture independent bacterial community profiles (stress value of 0.1616), D–F the cultured bacterial profiles (stress value of 0.3116), and G–I the PGP functional profiles of isolated bacterial cultures (stress value of 0.1762). Panel (A, D, G) shows samples grouped and colored by crop type, (B, E, H) by location, and (C, F, I) by soil type. Each point represents a sample, and colors correspond to the grouping variable shown in each panel, revealing patterns in community structure across environmental categories. Panels J–K show ANOSIM R values based on the data shown in plots A–I, respectively.

To analyse the variation of the community between samples, we applied non-metric multidimensional scaling (NMDS, Fig. 2A–C). Location was the primary driver of community variation (ANOSIM R = 0.936, *P* = .001, PERMANOVA F = 35.3425, R^2^ = 0.428, *P* = .001, Fig. 2B and J) followed by soil type (ANOSIM R = 0.4328, *P* = .001, PERMANOVA F = 22.4007, R^2^ = 0.181, *P* = .001, Fig. 2C and J) and crop was found to be the least important factor shaping community variation (ANOSIM R = 0.02727, *P* = .001, PERMANOVA F = 2.552, R^2^ = 0.03, *P* = .002, Fig. 2A and J). As such, according to the ANOSIM and PERMANOVA analyses, location was the main driver of community variation (ANOSIM R = 0.936), followed by soil type (ANOSIM R = 0.4328) and crop the least important driver (ANOSIM R = 0,02727) (Fig. 2J). This pattern was also observed using weighted-Unifrac distances and clr-normalized Aitchison distances (for more details see Supplementary Results, Figs S10a–c and S11a–c).

### Culture dependent bacterial community α-diversity

The alpha-diversity indices (Fig. 1  D, E, and F) showed that location, crop and soil type had a significant effect on Shannon’s H′ (Kruskal–Wallis *P* < .05, Fig. 1A, Fig. S3a–c) and S_obs_ (ANOVA *P* < .05 Fig. S3d–f) and DivNet estimated H′ (Fig. S8d–f  *P* < .0001), while no effect was observed in Pielou (Kruskal–Wallis *P* > .05, Fig. S3g–i). The diversity indices for S_obs_ and H′ showed a significant effect for location. However, pairwise tests identified slightly significant differences for location or soil type (Fig. S3  c, f, and  i) although it was significant for crop. The lowest S_obs_ (Fig. S3d and h′) (Fig. 1E) were observed in oats and sugar beet, while wheat showed the highest. Additionally, clay loam showed the lowest values for both indices (Fig. 1F, Fig. S3f). On the other hand, Faith’s PD only showed significant differences between crops (for more details see Supplementary Results, Fig. S7d).

### Culture dependent bacterial taxonomic community β-diversity

The NMDS plots obtained from the cultivated bacterial communities (Fig. 2D–F) showed more overlap between treatments than the culture independent bacterial communities (Fig. 2A–C). However, despite this overlap, samples obtained from the same location clustered together (Fig. 2E) (ANOSIM R = 0.2176, *P* = .001, PERMANOVA F = 4.0525, R^2^ = 0.1177, *P* = .001, Fig. 2K). Also, the biggest distance between samples from two locations was observed between Hertfordshire and Yorkshire-CY in both datasets. As in the culture independent analysis grouping based on crop was least apparent (Fig. 2D) (ANOSIM R = 0.06699, *P* = .001, PERMANOVA F = 2.237, R^2^ = 0.0519, *P* = .001, Fig. 2K). Additionally, the clustering based on soil type (Fig. 2F) was only clear for clay-rich samples (ANOSIM R = 0.1734, *P* = .001, PERMANOVA F = 4.9626, R^2^ = 0.0566, *P* = .001, Fig. 2K). These patterns were confirmed by ANOSIM and PERMANOVA. Both tests indicated that location had more influence on the β-diversity followed by soil type and crop (*P* = .001, Fig. 2K). Furthermore, the analysis based on weighted-Unifrac and clr-normalized Aitchison distances supported the finding that location was the main driver of differentiation (for more details see Supplementary Results, Figs S10d–f and S11d–f).

### Abundance of plant growth promoting traits in cultures

**Figure 3:**
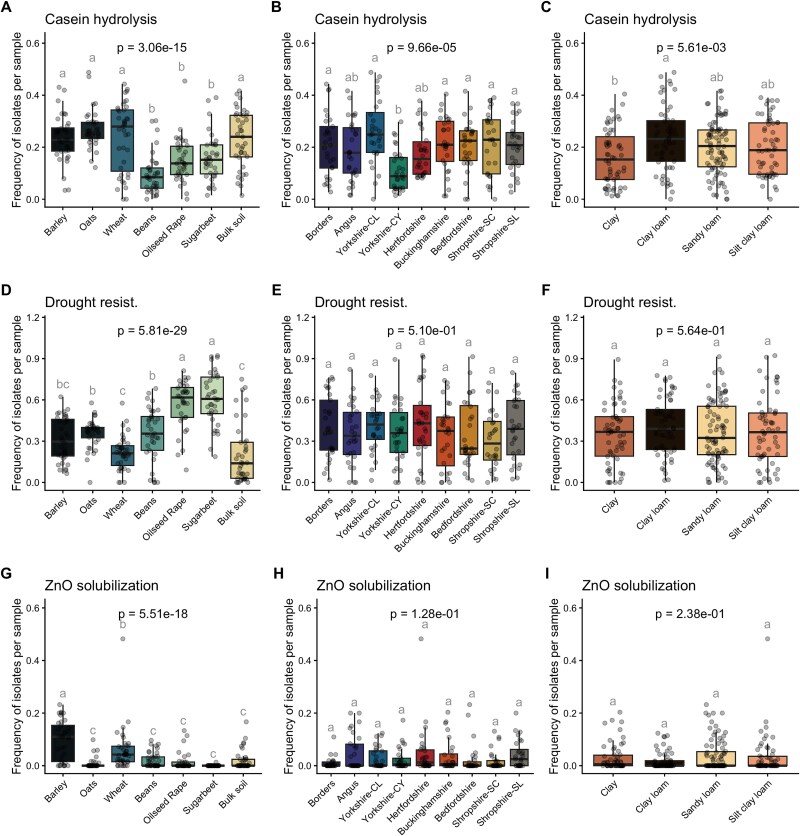
Frequency of bacterial cultures with PGP functions across environmental categories. Boxplots with overlaid jittered data points showing the distribution of frequencies of cultured microbes exhibiting distinct PGP functions. A, D, and G—Samples are grouped on crop type. B, E, and H—Samples are grouped on locations. C, F, and I—Samples are grouped on soil texture type. Boxes represent the IQR with medians indicated; whiskers extend to 1.5× IQR. Points represent individual observations. Samples are grouped according to soil type. Letters above the boxes indicate statistically significant groupings based on post hoc Tukey’s HSD test, and floating labels within each panel show the ANOVA *P*-value for that comparison.

The abundance of PGP characteristics varied significantly across locations (Fig. 3B, E, and  H, Fig. S5), soil type (Fig. 3C, F, and  I, Fig. S6), and crop type (Fig. 3A, D, and  G, Fig. S4). Among the specific PGP functions, Ca_3_(PO_4_)_2_ solubilizers were most abundant in samples from Angus (Fig. S5), cereals, bulk soil (Fig. S4), and sandy loam (Fig. S6). Potassium (K) solubilizers were predominantly found in bulk soil (Fig. S5). Siderophore-producing bacteria were enriched in sandy loam, bulk soil, and Angus (Figs S4 and S5). Casein hydrolysis activity was higher in Angus, cereals, bulk soil, and clay loam, and notably lower in bean rhizospheres (Fig. 3A–C, Figs S4 and S5). It was also found that ACC deaminase production was more abundant in bean-associated samples (Figs S4 and S5). Salt tolerance was elevated in cereal-rhizosphere samples, and Shropshire soil types (Figs S4 and S5), whereas drought resistance was highest in oilseed rape and sugar beet rhizospheres (Fig. 3D–F). Zinc solubilizers were enriched in barley-associated samples (Fig. 3G–I, Figs S4 and S5). In summary, each PGP function exhibited distinct patterns across these environmental factors, with all three treatments (location, soil type, and crop) significantly influencing PGP abundance. However, pairwise comparisons revealed that differences were more pronounced between crops than between soil types or locations. As such, crop type was the dominant factor in shaping microbiome function.

### Plant growth promoting cultures β-diversity

Discrimination of bacterial sample function from different locations was observed (Fig. 2H and L, ANOSIM R = 0.03378, *P* = .001, PERMANOVA F = 2.2356, R^2^ = 0.0668, *P* = .001). In addition, soil type showed a significant effect with PERMANOVA analysis (Fig. 2I and L, PERMANOVA F = 1.7532, R^2^ = 0.0202, *P* = .048), but not according to ANOSIM (R = 0.008761, *P* = .215). However, in contrast to the 16S rRNA gene data for both culture-dependent and independent analyses, crop type was the most important factor that determined rhizosphere microbiome function (Fig. 2G and L, ANOSIM R = 0.2437, *P* = .001, PERMANOVA F = 11.9667, R^2^ = 0.2217, *P* = .001). Thus, contrary to what was observed for the community composition, crop type is a more significant driver in shaping the β-diversity pattern of PGP functions (Fig. 2L) compared to location or soil type.

## Discussion

In this study, we utilized the UKCMB project resource to assess how environmental factors (location and soil type) and crop plant type influence key microbiome components: the overall bacterial community, readily culturable community, and PGP functions relating to nutrient cycling and abiotic stress tolerance. By using high-throughput methods to analyse the soil microbiota associated plant rhizospheres grown in controlled glasshouse experiments, we were able to disentangle these factors and reveal patterns that were not accessible by observing these facets separately. These results are of great importance in plant breeding and inoculant development because they shed light on how the microbiota of crop microbiomes are assembled in the rhizosphere. A clear understanding of bacterial establishment in the rhizosphere is of paramount importance for designing highly efficacious soil inoculants.

Our data shows that soil sample location was the most important factor shaping the bacterial community structure of culture independent and cultured bacteria in terms of taxonomic composition (Fig. 2A–F). Soil chemical and physical parameters are key established factors that determine community composition [33–36]. For example, soil compaction level determines the oxygen status of soil, which has a profound effect on the selection and function of microbial communities in this environment. In addition, nutrient status (e.g. carbon and nitrogen, macro and micronutrient bioavailability) [37, 38] plays an important role in determining the selective environment for microbiota in a given root microbiome. As such, both soil structure and nutrient status profoundly influence plant phenotype, including the production of root exudates [39] and root growth [40], which influences the selection of microbiota in the plant root environment. Furthermore, soil structure and nutrient status are influenced by crop management factors such as fertilization [23, 41], crop rotation [42] as well as tillage [43], and these processes also play an important role in the bacterial community assembly, which in turn impacts soil and root microbiome function.

We observed that abundance of each taxonomic group at class and order level (Figs S8–S13 and Tables S1–S6) varied according to location and soil type. However, the main taxonomic groups are highly abundant in all samples. For example, Acidobacteria, Actinobacteria, Bacilli, and Proteobacteria, are present in high abundance in most soils [33] and their relative contribution to the bacterial community varies depending on the conditions [34]. Actinobacteria are highly resilient bacteria often observed in soils under stress conditions and are associated with organic matter decomposition under such conditions [44, 45]. This could explain why they, especially, the Micrococcales, were found in high abundance using oligotrophic media (1/10^th^ TSA). On the other hand, Alphaproteobacteria and Bacilli are commonly highly abundant in nutrient rich soils [46] and rhizospheric soils [45]. Many isolates from these classes have been shown to promote plant growth and are commonly used as commercial inoculants [47]. One of the key highlights of this association is the enrichment of rhizobia in the fava beans rhizosphere since their natural association is important in more sustainable agricultural approaches [48]. Additionally, Verrucomicrobiae and Thermoleophilia are widespread bacteria that are recognized as oligotrophic organisms [49, 50]. Their increased abundance in the rhizosphere of fava beans and oats indicates that the root environment of these plants might not be as favorable for copiotrophs which are commonly associated with rhizospheres.

Selection of microbes in the rhizosphere of plants was categorized at two different levels in terms of importance. Although a given plant species selected specific taxa in their rhizospheres (Fig. 2A and D), this was a weaker selection compared to that imposed by the soil environment (Fig. 2B, C, E, and  F), as revealed by the PERMANOVA results. However, plant rhizospheres strongly selected microbes in terms of their PGP functions (Fig. 2G), and this selection was more pronounced than the shaping of PGP structure by soil type or soil location (Fig. 2H and I). As such, we found that bacterial plant growth promoting function was primarily selected by the plant species, whereas bacterial community taxonomy is shaped by soil type and location. Nonetheless, these functional results were obtained from in vitro tests, and their expression *in planta* may differ.

We have previously shown that rhizosphere soils select for PGP in the absence of chemical fertilizers [23, 51], hence utilizing the PGP potential of the local soil community when there is low abundance of labile nutrients. This relationship facilitates the plant’s access to nutrients, alleviates stress, and suppresses diseases [47]. It is, therefore, an advantage for the plants to foster the establishment of PGP rhizosphere populations and consequently increase their fitness in a low nutrient environment. However, modern plant breeding has led to a reduction in the capacity of crops to select for PGPR [23, 52, 53]. Nevertheless, our data indicates that, despite this reduction in plant-microbe communication, the functional selection of microbes by crops is still an important biological process when analysed using a multifaceted approach in elite modern crop varieties [52, 54].

Our data indicates that local soil characteristics act as the main driver for community assembly at a taxonomic level (ANOSIM R = 0.936) and that the selection of taxa exerted by the plant on these communities is minor (ANOSIM R = 0.2727). On the other hand, we found that the main selective pressure for bacterial function is plant species (ANOSIM R = 0.2437). Thus, there are different taxa capable of performing a given ecological function. This process might explain why we observe that differential taxa selection between crops is limited, whereas a high degree of species-dependent PGP functional selection exists. This is likely determined by the plant’s requirements or the selective environment created by a given plant root system [10]. Our observation that there was an increased abundance of microbes with tolerance to drought in oilseed rape and sugar beet is likely due to these crops having large tap roots compared with the more fibrous root systems of cereals and legumes. The large tap roots result in oilseed rape and sugar beet having a high-water demand, which could in turn create a rhizosphere environment with a lower water availability which selects for microbiota with high drought tolerance [10]. In such conditions, bacteria that are capable of producing extracellular polysaccharides or osmoprotectants may be better equipped to resist the lower water availability [47]. In addition, our observation that N hydrolyzation was significantly lower in the rhizosphere of beans, could imply that there is a reduced demand for N acquisition via this route in the legume rhizosphere due to the establishment of the *Rhizobium* mutualism providing legumes with an adequate supply of this element. Furthermore, we observed an increased selection of Zn solubilizing microbes in the barley rhizosphere. Although barley Zn content is not especially high compared to other crops, this crop is vulnerable to low Zn levels in soil [55], thus favoring a positive selection for Zn solubilizing microbes in the rhizosphere. These bacteria often produce organic acids such as oxalic acid and gluconic acid that solubilizes Zn through local pH alteration [56]. As such, the differences we found in microbiome function in the rhizosphere reflect the requirements of the given crop, whereas taxonomic status of soil microbiomes are less relevant in terms of bacterial function, presumably due to high functional redundancy of microbes in soil [57]. Plants produce root exudates that stimulate the soil community and shape the rhizosphere [58]. Most of these molecules act as nutrient sources and lead to the growth of certain populations with greater fitness to make use of these nutrients [59]. A subset of the bacterial populations found in the rhizosphere carry genes to that make use of these [60] exudate molecules as signaling molecules that attract microbes to the rhizosphere, resulting in changes of rhizosphere community composition [59]. Strigolactones, flavonoids, and organic acids are examples of molecules that induce a response from arbuscular mycorrhiza, *Rhizobium*, and *Bacillus subtilis*, respectively [9, 59].

The relationship between plant–microbiome–soil and its effect on the microbial community structure will have an important role in the establishment of microbial populations in the rhizosphere. This establishment is a key part of the development of microbial inoculants for agricultural use [10]. A microbial inoculant must colonize its host plant, survive, and persist in the environment, while exercising its intended function [61]. Our data indicates that soils have an important effect on the community’s composition, while function was less affected. This suggests that a “one size fits all” strategy for microbial inoculation may not be as effective as a bespoke approach that takes into consideration the soil characteristics and inoculant niche breadth.

We found that plants exert a strong effect on the function of their rhizosphere community. The selection of varieties for productivity since the green revolution has, inadvertently, led to a decrease in PGPRs [23, 51]. Consequently, our data support the idea that breeding strategies should consider their effect on the PGPR community to ensure that their ecological services remain active, which could in turn reduce input costs and increase sustainability. This finding has implications for the delivery of microbiome facilitated agriculture and may help prioritize whether inoculation or plant breeding/gene editing approaches should be adopted. Due to the high functional redundancy of soil microbiomes, it may be the case that the best strategy for successful microbial contribution to enhanced plant nutrition will come through selection of the locally adapted microbes through plant modification, as opposed to inoculation of non-cognate microbes, which must overcome challenge from the indigenous community in order to successfully establish in the plant environment.

The main outcome from our work is the discovery that bacterial function is selected by plant type irrespective of edaphic and geographical conditions. Conversely, the observation that soil type and sample location are most significant for taxonomic structuring of the rhizosphere microbiota indicates that soils harbor a high degree of functional redundancy. This in turn allows plants to select populations from the local community based on their functional traits [61]. Our results show that the interplay between plant selection and environmental conditions is responsible (at different levels) for the structure and function of the bacterial community. By integrating high-throughput culture-dependent and culture-independent approaches, alongside functional screening of readily culturable microbes, we revealed both redundancy and specificity in bacterial community assembly and activity, respectively. Importantly, we show that although certain bacterial taxa are widespread, their functional traits vary significantly with environmental context, emphasizing the need to consider both taxonomy and function in microbiome-based applications. However, the functional data presented here was obtained from culturable bacteria with a single medium, which we acknowledge represents a small proportion of the microbial community [62, 63]. Thus, future work using meta-genomic/transcriptomic approaches as well as the use of different culture media to capture a broader range of the microbiome, including other Kingdoms such as Fungi, could help elucidate mechanisms that are important for nutrient cycling, abiotic stress tolerance and also for other processes such as the production of secondary metabolites relevant for the suppression of plant pathogens.

To conclude, in this study we leveraged the UKCMCB, a uniquely rich resource, to explore these relationships which support the development of tailored microbial solutions for sustainable agriculture. Our findings show that bacterial function is primarily determined by plant type, irrespective of edaphic and geographical conditions, whereas community composition is shaped by soil legacy. Altogether, these results provide a foundation for developing and applying bacterial inoculants based on ecological understanding, which can enhance inoculation success, improve crop productivity, and promote soil sustainability.

## Supplementary Material

Suppl_Infor_R2_ycag083

## Data Availability

The datasets generated during and/or analysed during the current study are available in ENA database on the bioproject PRJEB58189 (https://www.ebi.ac.uk/ena/browser/view/ PRJEB58189).
