# Supplementary material for: Host plant selects bacterial rhizosphere microbiome function whereas community structure is determined by soil legacy
Source: ISME Commun. 2026 Mar 28;6(1):ycag083. doi: 10.1093/ismeco/ycag083 (PMC13137329; doi:10.1093/ismeco/ycag083)
Supplement: Suppl_Infor_R2_ycag083 [file suppl_infor_r2_ycag083.pdf]

Supplementary Material

Host plant selects bacterial rhizosphere microbiome function whereas community structure is determined by soil legacy

Taketani, R. G.[1], Clark, I.M.[1], Yau, P.T.O.[2], Liu, L.[1], Zhang, F.[1], Bak, G.R.[1], Thompson C.M.A.[3], Bonnin, J.M.[4], Stewart, H.[4] Malone, J.G.[3,5], Jones, S.[6], Holden, N.[2], Ryan, M.J.[4] and Mauchline T.H.[1]

1. Rothamsted Research, West Common, Harpenden, Hertfordshire AL5 2JQ
2. Scotland's Rural College (SRUC), AB21 9YA Aberdeen, UK
3. John Innes Centre, Norwich Research Park, Colney Ln, Norwich NR4 7UH, UK
4. CABI, Silwood Park, Buckhurst Road, Ascot, SL5 7PY
5. School of Biological Sciences, University of East Anglia, Norwich NR4 7TJ, UK
6. Information and Computational Sciences, The James Hutton Institute, Invergowrie, Dundee DD2 5DA, UK

Supplementary Figures

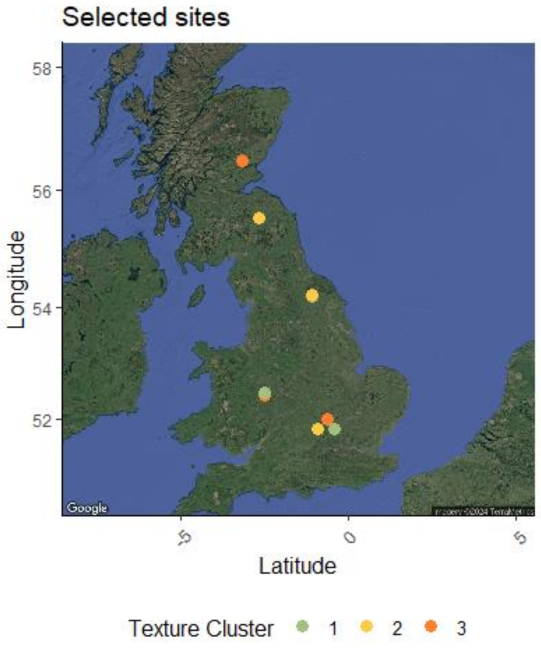

Figure S1: Map of the sampling locations. Each dot corresponds to one sampled field. Dots are coloured according to complete linkage clusters based on their texture data. The two samples from Yorkshire (northeast) are overlapped they represent Texture Clusters 1 and 2, the only point visible is Yellow.

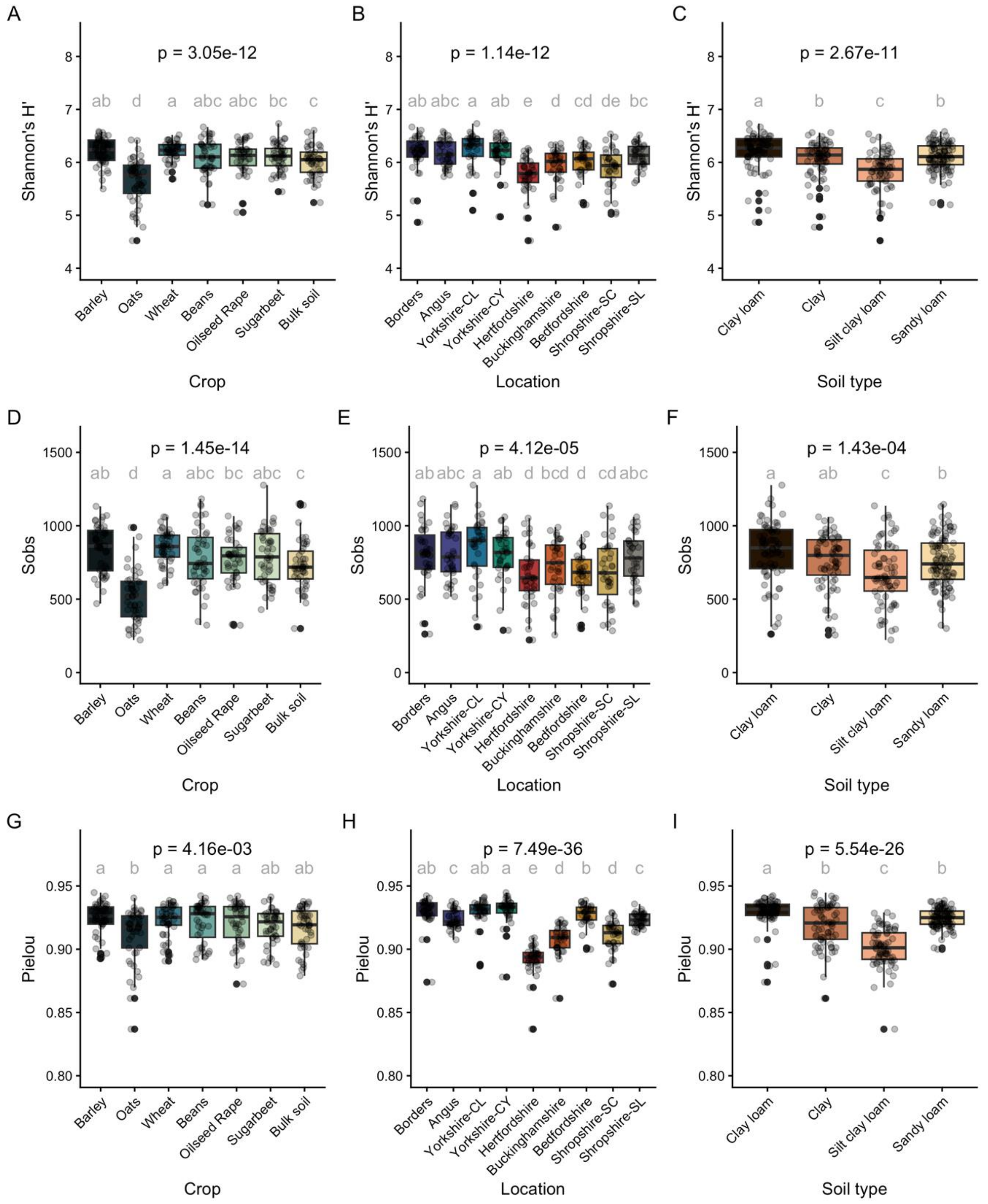

Figure S2: Alpha-diversity metrics based on 16S rRNA gene amplicon sequence variants obtained from culture independent bacterial communities. A, B, C – Shannon's H' diversity index. D, E, F – Sobs (observed ASVs). G, H, I – Pielou's evenness index. A, D, G – diversity index grouped on locations. B, E, H – diversity index grouped on crops. C, F, I – diversity index based on soil texture type. Boxplots show the distribution of diversity indexes across groups, with the central line representing the median, the box indicating the interquartile range (IQR), and whiskers extending to 1.5×IQR. Each dot represents the diversity index for a sample. Treatments were compared with Kruskal-Wallis test followed by pairwise Wilcoxon rank sum exact test with Benjamin-Hochberg p-value adjustment. Letters above boxes represent the groups formed by Wilcoxon test.

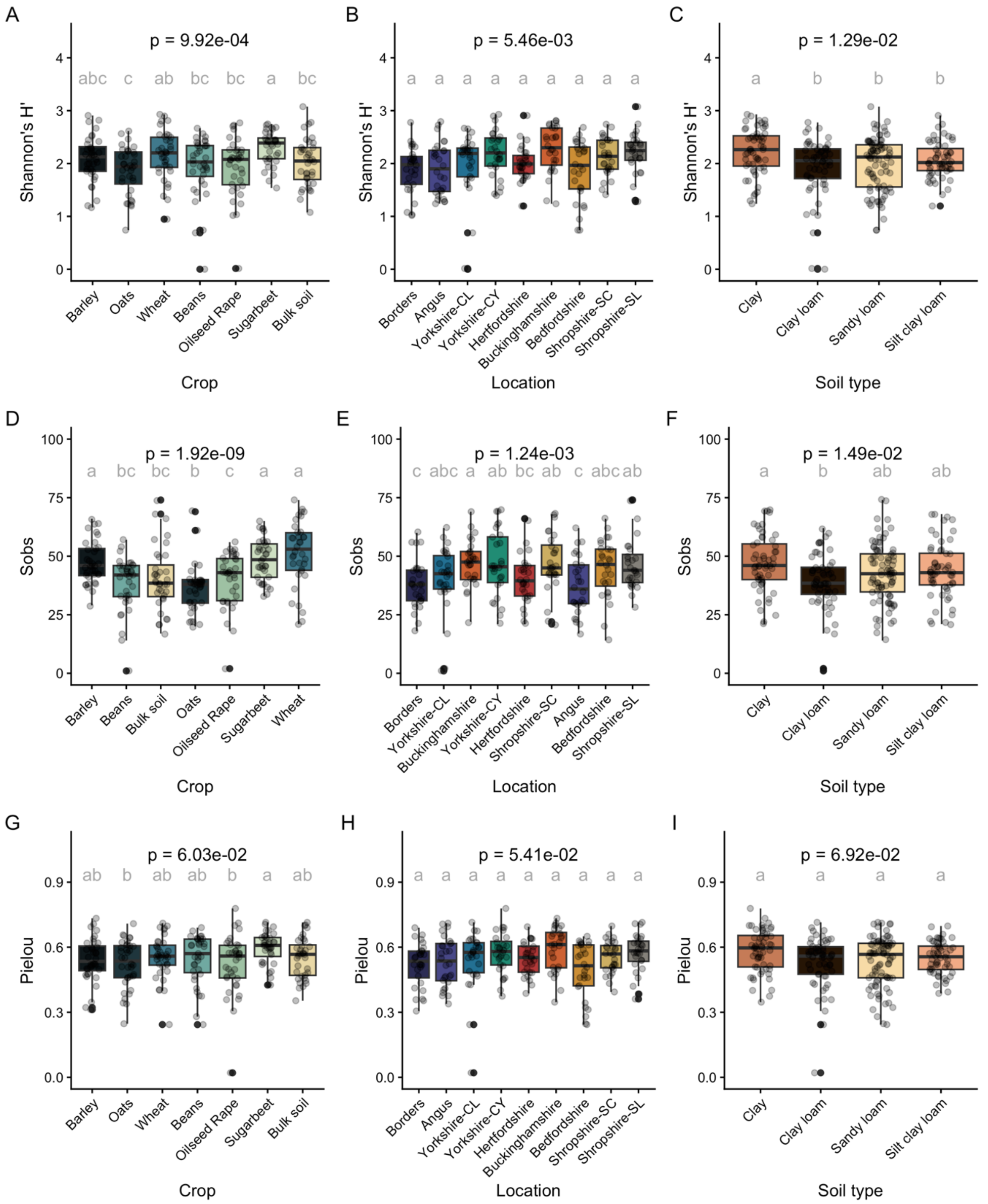

Figure S3: Alpha-diversity metrics based on 16S rRNA gene amplicon sequence variants obtained from cultured bacterial communities. A, B, C – Shannon's H' diversity index. D, E, F – Sobs (observed ASVs). G, H, I – Pielou's evenness index. A, D, G – diversity index grouped on locations. B, E, H – diversity index grouped on crops. C, F, I – diversity index based on soil texture type. Boxplots show the distribution of diversity indexes across groups, with the central line representing the median, the box indicating the interquartile range (IQR), and whiskers extending to 1.5×IQR. Each dot represents the diversity index for a sample. Treatments were compared with Kruskal-Wallis test followed by pairwise Wilcoxon rank sum exact test with Benjamin-Hochberg p-value adjustment. Letters above boxes represent the groups formed by Wilcoxon test.

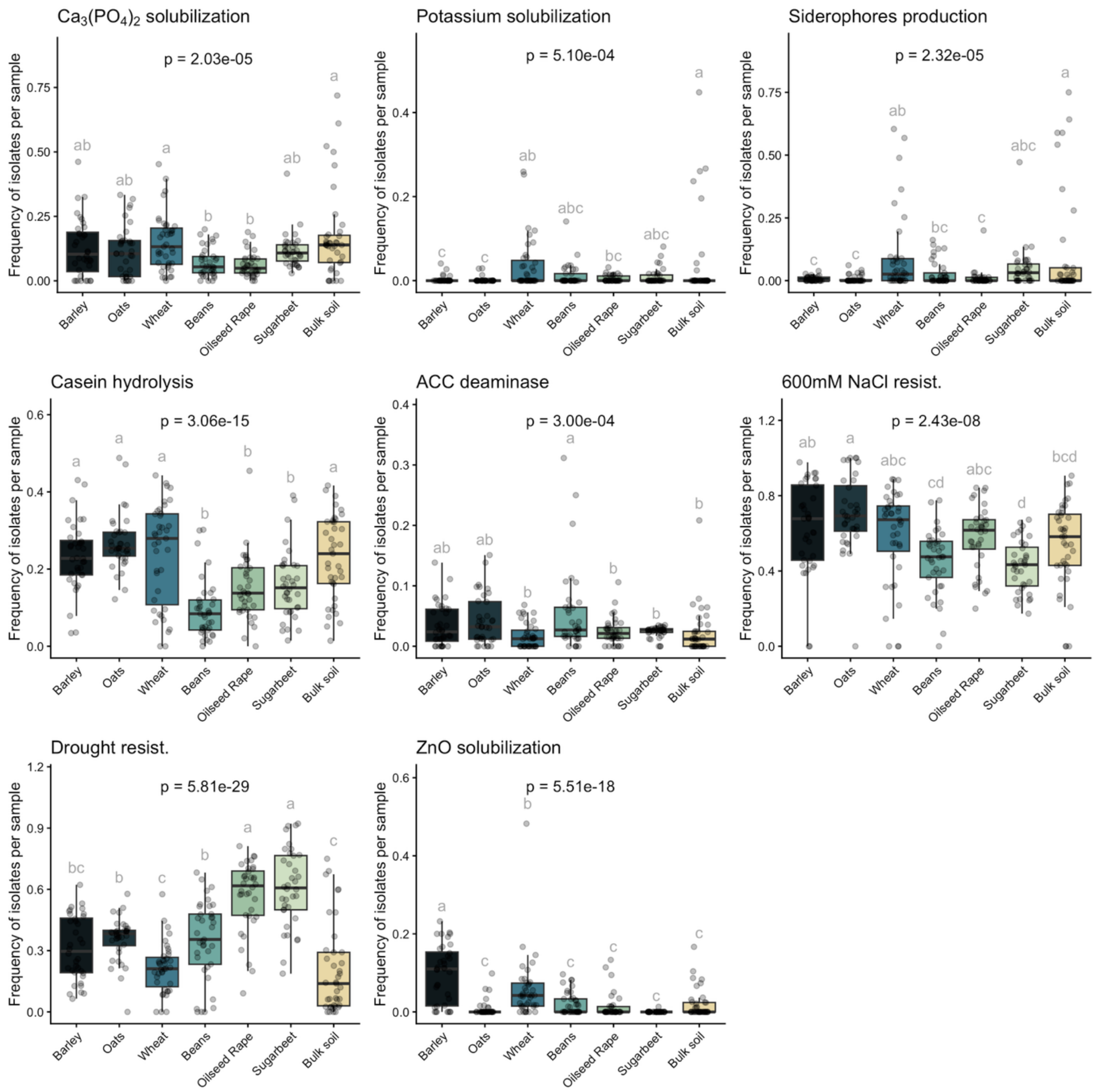

Figure S4: Frequency of microbial cultures with plant growth-promoting (PGP) functions across different crops. Boxplots with overlaid jittered data points showing the distribution of frequencies of cultured microbes exhibiting distinct PGP functions. Each panel represents a different PGP trait (e.g., phosphate solubilisation, siderophore production, etc.). Boxes represent the interquartile range (IQR) with medians indicated; whiskers extend to 1.5× IQR. Points represent individual observations. Samples are grouped according to crop. Letters above the boxes indicate statistically significant groupings based on *post hoc* Tukey's HSD test, and floating labels within each panel show the ANOVA p-value for that comparison.

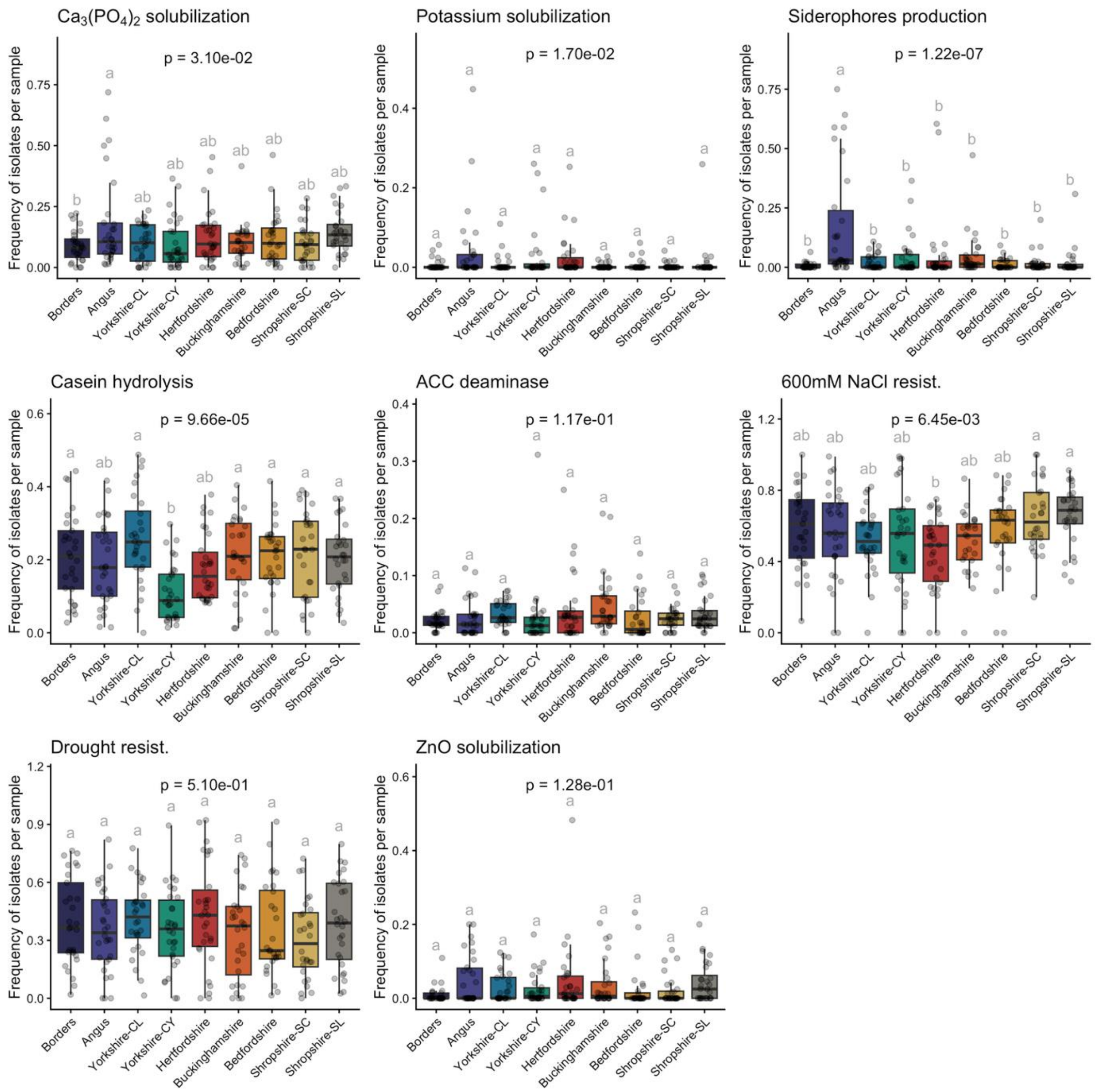

Figure S5: Frequency of microbial cultures with plant growth-promoting (PGP) functions across different locations. Boxplots with overlaid jittered data points showing the distribution of frequencies of cultured microbes exhibiting distinct PGP functions. Each panel represents a different PGP trait (e.g., phosphate solubilisation, siderophore production, etc.). Boxes represent the interquartile range (IQR) with medians indicated; whiskers extend to 1.5× IQR. Points represent individual observations. Samples are grouped according to location. Letters above the boxes indicate statistically significant groupings based on *post hoc* Tukey's HSD test, and floating labels within each panel show the ANOVA p-value for that comparison.

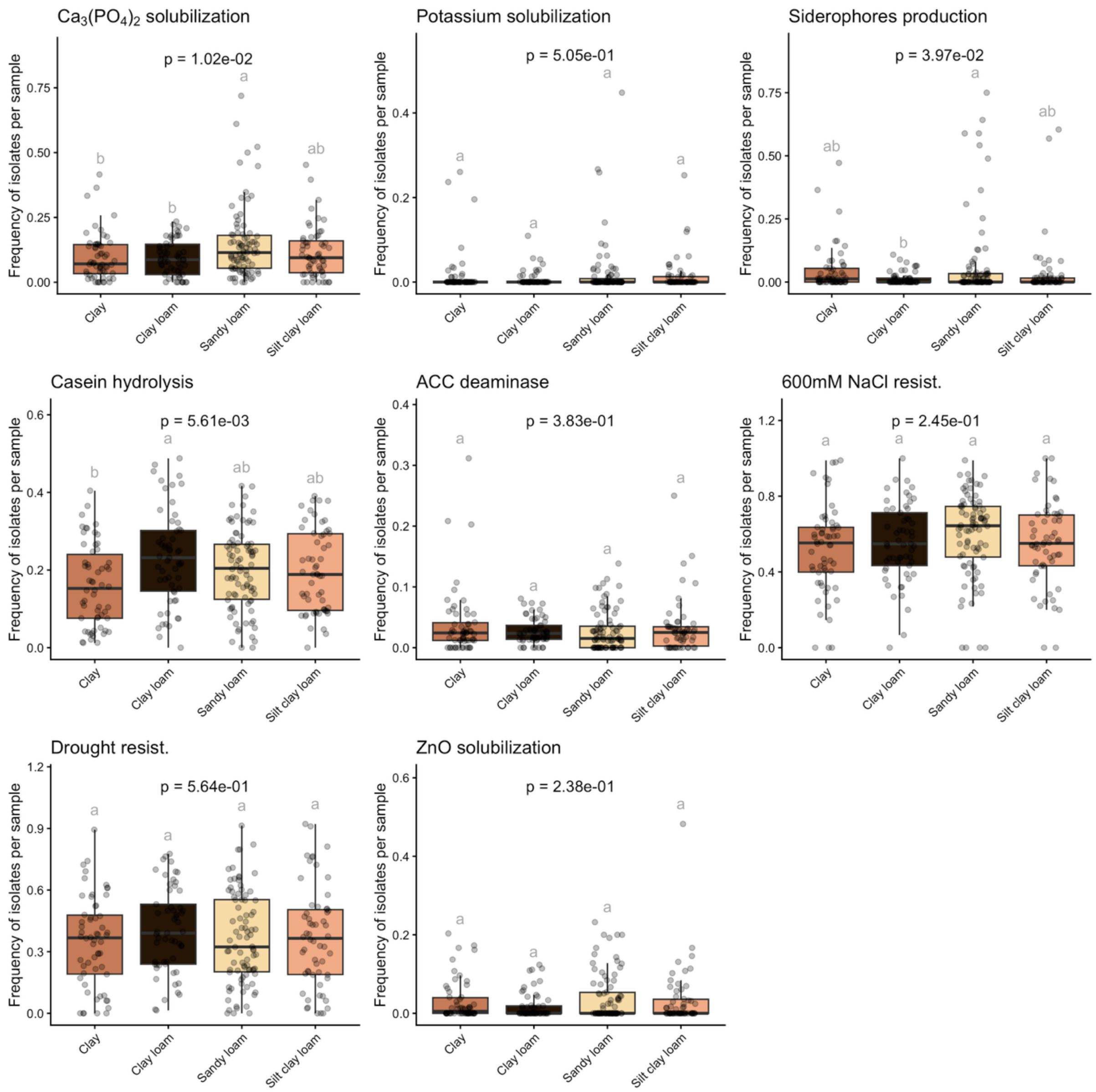

Figure S6: Frequency of microbial cultures with plant growth-promoting (PGP) functions across different soil types. Boxplots with overlaid jittered data points showing the distribution of frequencies of cultured microbes exhibiting distinct PGP functions. Each panel represents a different PGP trait (e.g., phosphate solubilisation, siderophore production, etc.). Boxes represent the interquartile range (IQR) with medians indicated; whiskers extend to 1.5× IQR. Points represent individual observations. Samples are grouped according to soil type. Letters above the boxes indicate statistically significant groupings based on *post hoc* Tukey's HSD test, and floating labels within each panel show the ANOVA p-value for that comparison.

Supplementary Materials and Methods

Plant Growth Promoting High-throughput Tests – Media

The PGP functions were all accessed using solid media. The cultures were incubated at room temperature (22-25°C) for up to 7 days. The details of each assay are as follows:

Phosphorus solubilization: To evaluate the cultures capacity to solubilise phosphate, the cultures were inoculated in Pikovskaya agar (Pikovskaya, 1948) containing tricalcium phosphate (0.05% yeast extract, 1% D-glucose, 0.5% $Ca_3(PO_4)_2$, 0.05% $(NH_4)_2SO_4$, 0.02% KCl, 0.01% $MgSO_4{\bullet}7H_2O$, 0.00001%

$MnSO_4 \cdot H_2O$, 0.00001% $FeSO_4 \cdot 7H_2O$, 1.5% agar). Positive cultures produced a clear halo on the opaque media.

Potassium solubilization: The cultures were inoculated on Aleksandrow agar plates (Aleksandrov et al., 1967) containing potash feldspar (0.05% $MgSO_4 \cdot 7H_2O$, 0.01% $CaCO_3$, 0.2% potash feldspar, 0.5% D-glucose, 0.0005% $FeCl_3 \cdot 6H_2O$, 0.2% $Ca_3(PO_4)_2$, 2% agar, pH 7.0–7.2). Positive cultures produced a clear halo on the opaque media.

Casein hydrolysis: Casein agar (Frazier and Rupp 1928) was used to access the culture's capacity to hydrolyse organic nitrogen. The media contained 5% skimmed milk powder, 0.5% pancreatic digest of casein, 0.25% yeast extract, 0.1% D-glucose, 1.25% agar. Positive cultures produced a clear halo on the opaque media.

Siderophore production: Media containing chrome azurol S (CAS) (Louden et al., 2011; Schwyn and Neilands, 1987) was used to access siderophore production by the cultures. The media preparation followed Louden et al., 2011 protocol. Positive isolates were determined from the presence of an orange halo surrounding the colony.

Zinc solubilisation: Zinc solubilising agar (Subba Rao, 1977) was used to demonstrate the cultures capacity to solubilise ZnO. The media formulation contained 1% D-glucose, 0.1% $(NH_4)_2SO_4$, 0.02% KCl, 0.01% K_2HPO_4, 0.02% $MgSO_4 \cdot 7H_2O$, 0.1% ZnO, 1.5% agar. Positive cultures produced a clear halo on the opaque media.

ACC deaminase production: ACC deaminase activity was tested according to Penrose and Glick, (2003). Briefly, the cultures were inoculated onto three different media; M9 (0.6% Na_2HPO_4, 0.3% KH_2PO_4, 0.05% NaCl, 0.1% NH_4Cl, 0.05% glucose, 0.05% mannitol, 0.05% arabinose, 0.05% trehalose, 0.0011% $CaCl_2$, 0.024% $MgSO_4$), M9 without NH_4Cl and M9 without NH_4Cl but supplemented with 30 μM of ACC as nitrogen source. The growth was compared between the three media and considered positive when the culture only grew in M9 with ACC.

Salt stress resistance: Cultures were inoculated in 10% Tryptone Soya Agar (TSA) with the addition of 600mM NaCl. Cultures capable of growth in such concentration of NaCl were considered resistant to salt stress.

Drought tolerance: Cultures were inoculated in 10% Tryptone Soya Agar (TSA) with the addition of 285g/L sorbitol to test their capacity to tolerate low water availability. Cultures capable of growth in such concentration of sorbitol were considered resistant to drought.

Supplementary Results

Culture independent bacterial community phylogenetic α-diversity

The normalised ASV table served as the basis for calculating Faith's alpha-diversity metrics (Figs. 1A–C; Fig. S1). Diversity indices varied significantly across groups for all assessed factors, location, crop, and soil type ($p < 0.001$). Among locations, the northernmost sites (Borders, Angus, Yorkshire-CL and CY) exhibited higher diversity (n = 35) (Fig. S7B), whereas the Hertfordshire site showed markedly lower values. Across crops, oat samples displayed significantly reduced diversity and richness compared to the other crop types (n = 45) (Fig. S7A). For soil type, clay loam had the highest diversity and richness, while silty clay loam showed the lowest values (Fig. S7C).

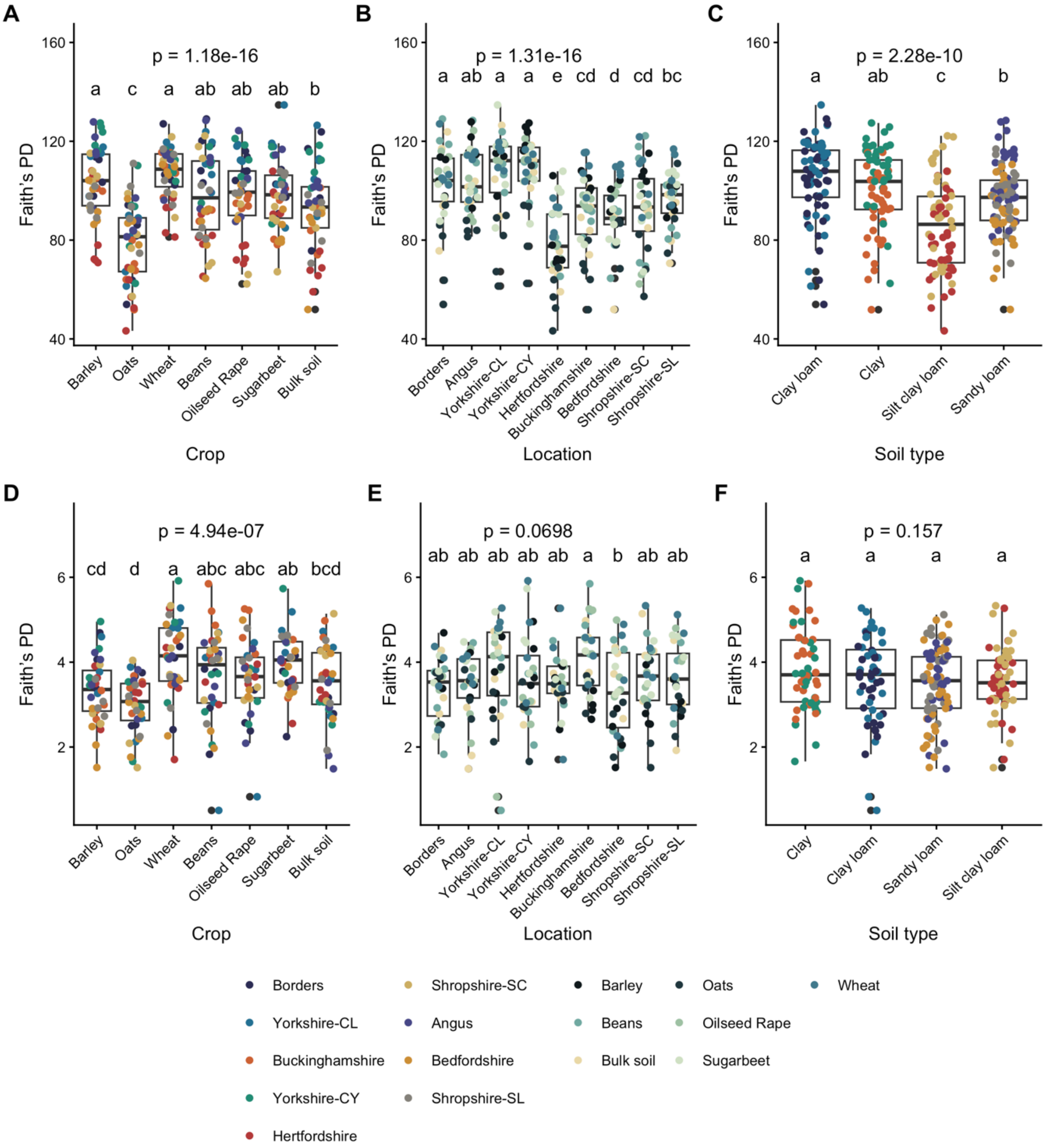

Figure S7: Faith's PD alpha-diversity metrics based on 16S rRNA gene amplicon sequence variants (ASVs). Panels A-C show the culture independent bacterial community profiles, D-F the cultured bacterial profiles. A, D – diversity index grouped on crop type, n=45 and 36 respectively. B, E – diversity index grouped on locations, n=35 and 28 respectively. C, F – diversity index based on soil texture type, n=70 and 56 respectively (except for sandy loam n=105 and 84 respectively). Boxplots show the distribution of diversity indexes across groups, with the central line representing the median, the box indicating the interquartile range (IQR), and whiskers extending to 1.5×IQR. Each dot represents the diversity index for a sample and are coloured according to: A, C, D, and F – locations, and B and E – crop, following the legend. Plots B and C were compared with Kruskal-Wallis test followed by pairwise Wilcoxon rank sum exact test and plots A, D, E, and F were analysed using ANOVA followed by Tukey's *post-hoc* test. Letters above boxes represent the groups formed by Wilcoxon and Tukey's tests.

The non-rarefied ASV table was used to estimate the Shannon’s H’ diversity index using DivNet (Fig. S8A-C). The pattern observed for this estimated index was similar to the other indexes with silt clay loam, Oats, and Hertfordshire showing a lower diversity while clay loam, wheat, and Yorkshire-CY higher (Fig S8A-C) this pattern was confirmed with *betta* ($p < 0.0001$).

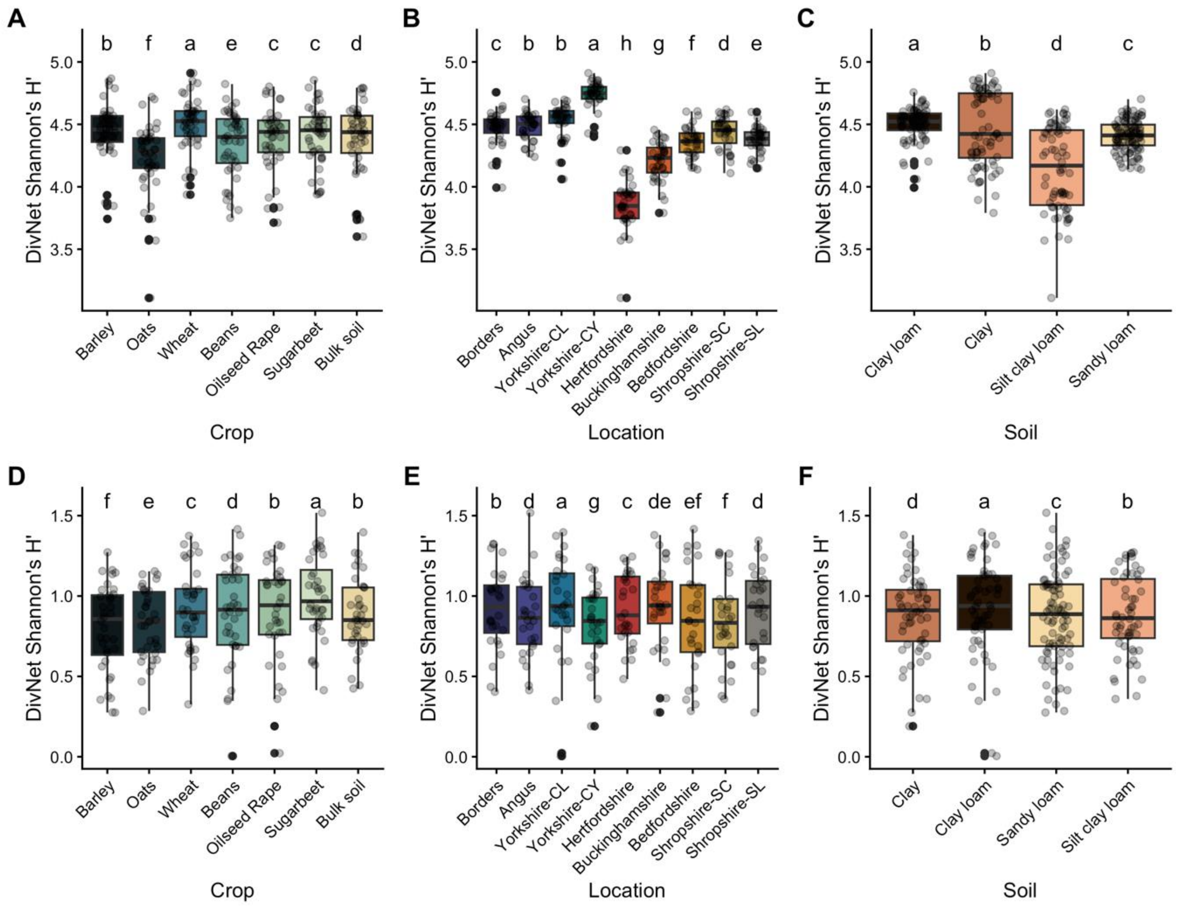

Figure S8: DivNet Shannon’s H’ estimates based on 16S rRNA gene amplicon sequence variants (ASVs). Panels A-C show the culture independent bacterial community profiles, D-F the cultured bacterial profiles. A, D – diversity index grouped on crop type, n=45 and 36 respectively. B, E – diversity index grouped on locations, n=35 and 28 respectively. C, F – diversity index based on soil texture type, n=70 and 56 respectively (except for sandy loam n=105 and 84 respectively). Boxplots show the distribution of diversity indexes across groups, with the central line representing the median, the box indicating the interquartile range (IQR), and whiskers extending to 1.5×IQR. Each dot represents the diversity index for a sample and are coloured according to: A, C, D, and F – locations, and B and E – crop, following the legend.

Culture independent bacteria taxonomy

In all soils analysed, it was found that there was a high abundance of Actinobacteria, Alphaproteobacteria, Bacilli, Thermoleophilia, and Verrucomicrobiae. Together, these classes represented about 50% of the community (Fig. S9B, Table S2). The sample from Hertfordshire showed a higher abundance of Bacilli (2-4 fold) and Actinobacteria compared to the other locations and consequently there was a reduction in relative abundance of Acidobacteria, Bacteroidia,

Vicinamibacteria, and other minor classes in this soil compared to other soil samples. In addition, in the samples from Buckinghamshire, a decrease in abundance of minor classes such as Clostridia and Gemmatimonadetes was observed due to a higher abundance of the dominant classes mentioned above. Although the differences observed in the other samples were not as large as the two examples described, none of the locations shared the same taxonomic profile.

The changes observed between samples obtained from different crops were smaller than those observed for location and more evident in the dominant classes (Fig. S9A, Table S1). Verrucomicrobiae were more abundant in oats and bean rhizospheres while less abundant in bulk soil compared to the other crops. Thermoleophilia had its lowest abundance in wheat while it was most abundant in oat rhizospheres. Alphaproteobacteria was more abundant in bulk soil and sugar beet rhizosphere, and Actinobacteria in barley and bulk soil. Thus, all the crops had different taxonomic profiles despite sharing the same dominant classes.

Different soil types changed the taxonomic profile of the community (Fig. S9C, Table S3). The profiles from clay loam and sandy loam were similar to each other in comparison to the other soil types. The silt clay loams had the most different taxonomic composition due to the higher abundance of Bacilli and Actinobacteria and lower abundance of Vicinamibacteria. Similarly, the higher abundance of Bacteroidia observed in the clay samples was due to Buckinghamshire samples being part of this group.

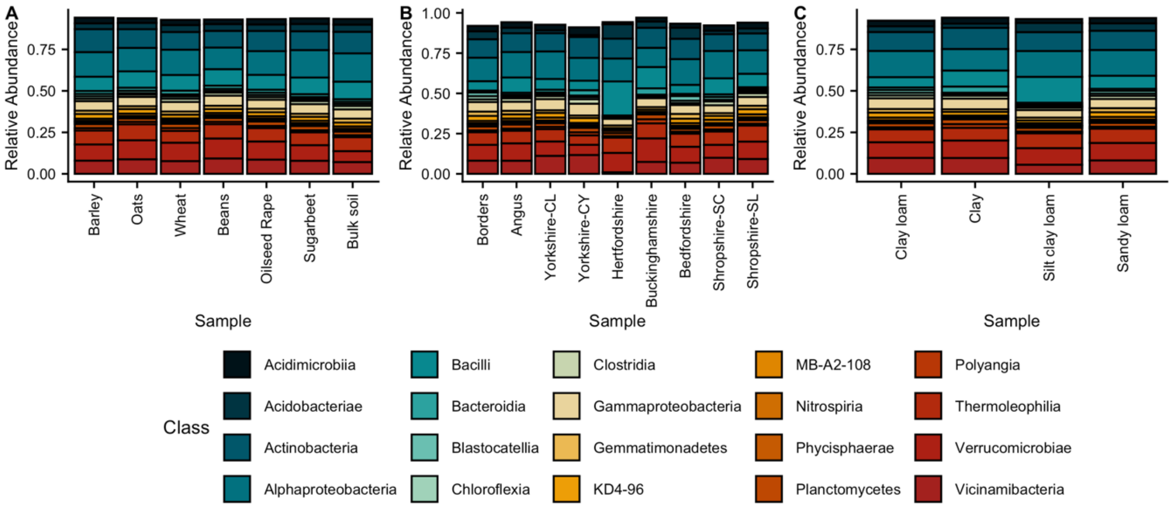

Figure S9: Taxonomic composition of culture independent bacterial ASVs across samples grouped by environmental variables. Stacked bar charts showing the relative abundance of culture independent bacterial amplicon sequence variants (ASVs) derived from 16S rRNA gene sequencing, assigned at the taxonomic level of class. Bars are coloured according to taxonomic class affiliation. Panel (A) shows samples grouped by crop type, (B) by location, and (C) by soil type. Each bar represents the composite microbial community profile for the respective grouping.

Table S1: Frequency of Culture Independent Bacterial Classes per Crop type.

Class	Barley	Beans	Bulk soil	Oats	Oilseed rape	Sugarbeet	Wheat
**Acidimicrobiia**	0.035 ± 0.010(b)	0.027 ± 0.009(a)	0.035 ± 0.014(b)	0.028 ± 0.013(ab)	0.032 ± 0.012(ab)	0.034 ± 0.011(ab)	0.032 ± 0.009(ab)
**Acidobacteriae**	0.035 ± 0.020(a)	0.041 ± 0.027(a)	0.043 ± 0.029(a)	0.038 ± 0.028(a)	0.039 ± 0.029(a)	0.044 ± 0.033(a)	0.042 ± 0.024(a)
**Actinobacteria**	0.138 ± 0.025(c)	0.101 ± 0.016(a)	0.130 ± 0.026(bc)	0.113 ± 0.022(ad)	0.122 ± 0.026(bd)	0.114 ± 0.028(ad)	0.107 ± 0.013(a)
**Alphaproteobacteria**	0.149 ± 0.018(c)	0.131 ± 0.019(a)	0.171 ± 0.030(b)	0.141 ± 0.023(ac)	0.143 ± 0.020(ac)	0.166 ± 0.023(bd)	0.153 ± 0.023(cd)
**Bacilli**	0.086 ± 0.051(a)	0.099 ± 0.051(a)	0.106 ± 0.065(a)	0.099 ± 0.058(a)	0.090 ± 0.046(a)	0.101 ± 0.049(a)	0.095 ± 0.039(a)
**Bacteroidia**	0.018 ± 0.012(a)	0.019 ± 0.011(a)	0.015 ± 0.008(a)	0.019 ± 0.015(a)	0.018 ± 0.011(a)	0.017 ± 0.009(a)	0.022 ± 0.014(a)
**Blastocatellia**	0.014 ± 0.010(a)	0.013 ± 0.009(a)	0.013 ± 0.010(a)	0.015 ± 0.011(a)	0.014 ± 0.011(a)	0.012 ± 0.008(a)	0.012 ± 0.008(a)
**Chloroflexia**	0.014 ± 0.006(c)	0.010 ± 0.006(ab)	0.012 ± 0.006(ac)	0.008 ± 0.004(b)	0.013 ± 0.006(ac)	0.012 ± 0.006(ac)	0.014 ± 0.006(c)
**Clostridia**	0.015 ± 0.006(ab)	0.018 ± 0.008(ab)	0.020 ± 0.009(a)	0.014 ± 0.007(b)	0.015 ± 0.009(ab)	0.019 ± 0.011(ab)	0.019 ± 0.009(ab)
**Gammaproteobacteria**	0.057 ± 0.011(a)	0.061 ± 0.013(a)	0.056 ± 0.012(a)	0.056 ± 0.014(a)	0.054 ± 0.009(a)	0.059 ± 0.012(a)	0.058 ± 0.012(a)
**Gemmatimonadetes**	0.020 ± 0.007(ab)	0.020 ± 0.006(ab)	0.024 ± 0.008(a)	0.017 ± 0.008(b)	0.021 ± 0.008(ab)	0.018 ± 0.005(b)	0.019 ± 0.006(b)
**KD4-96**	0.028 ± 0.010(a)	0.024 ± 0.009(a)	0.023 ± 0.009(a)	0.028 ± 0.011(a)	0.024 ± 0.010(a)	0.024 ± 0.008(a)	0.023 ± 0.007(a)
**MB-A2-108**	0.013 ± 0.007(a)	0.013 ± 0.007(a)	0.014 ± 0.008(a)	0.014 ± 0.008(a)	0.014 ± 0.007(a)	0.014 ± 0.008(a)	0.012 ± 0.006(a)
**Nitrospiria**	0.007 ± 0.003(ab)	0.008 ± 0.003(ab)	0.008 ± 0.004(ab)	0.006 ± 0.003(a)	0.007 ± 0.003(ab)	0.008 ± 0.004(ab)	0.009 ± 0.004(b)
**Phycisphaerae**	0.012 ± 0.005(c)	0.009 ± 0.005(abc)	0.008 ± 0.004(ab)	0.008 ± 0.005(b)	0.011 ± 0.004(ac)	0.009 ± 0.004(abc)	0.011 ± 0.004(c)
**Planctomycetes**	0.025 ± 0.007(abc)	0.024 ± 0.007(abc)	0.022 ± 0.007(ab)	0.021 ± 0.008(b)	0.027 ± 0.008(c)	0.026 ± 0.006(ac)	0.026 ± 0.008(ac)
**Polyangia**	0.014 ± 0.006(ab)	0.014 ± 0.005(ab)	0.012 ± 0.007(a)	0.015 ± 0.007(ab)	0.012 ± 0.006(a)	0.012 ± 0.005(a)	0.017 ± 0.008(b)
**Thermoleophilia**	0.084 ± 0.014(a)	0.086 ± 0.019(ab)	0.084 ± 0.018(a)	0.096 ± 0.018(b)	0.081 ± 0.014(ac)	0.077 ± 0.017(ac)	0.072 ± 0.016(c)
**Verrucomicrobiae**	0.098 ± 0.026(cd)	0.121 ± 0.028(a)	0.065 ± 0.025(b)	0.115 ± 0.026(ac)	0.110 ± 0.029(acd)	0.095 ± 0.037(d)	0.111 ± 0.023(acd)
**Vicinamibacteria**	0.079 ± 0.031(a)	0.092 ± 0.040(a)	0.071 ± 0.033(a)	0.087 ± 0.037(a)	0.085 ± 0.039(a)	0.078 ± 0.031(a)	0.076 ± 0.027(a)

Table S1: Relative abundance of classes. Values are presented as mean ± standard deviation (SD) (with Tukey group letters).

Table S2: Frequency of Culture Independent Bacterial Classes per Location

Class	Borders	Yorkshire-CL	Buckinghamshire	Yorkshire-CY	Hertfordshire	Shropshire-SC	Angus	Bedfordshire	Shropshire-SL
**Acidimicrobiia**	0.034 ± 0.006(a)	0.035 ± 0.007(a)	0.024 ± 0.008(b)	0.049 ± 0.008(c)	0.014 ± 0.005(d)	0.034 ± 0.008(a)	0.035 ± 0.006(a)	0.025 ± 0.006(b)	0.037 ± 0.007(a)
**Acidobacteriae**	0.049 ± 0.010(f)	0.018 ± 0.006(a)	0.039 ± 0.010(b)	0.011 ± 0.004(a)	0.089 ± 0.018(c)	0.021 ± 0.015(ad)	0.034 ± 0.008(b)	0.069 ± 0.022(e)	0.030 ± 0.013(bd)
**Actinobacteria**	0.115 ± 0.017(abc)	0.113 ± 0.023(abc)	0.125 ± 0.028(a)	0.130 ± 0.019(a)	0.123 ± 0.031(ab)	0.104 ± 0.021(c)	0.116 ± 0.030(abc)	0.126 ± 0.024(a)	0.105 ± 0.023(bc)
**Alphaproteobacteria**	0.147 ± 0.021(cd)	0.171 ± 0.024(a)	0.119 ± 0.017(b)	0.142 ± 0.022(c)	0.142 ± 0.018(c)	0.170 ± 0.024(a)	0.159 ± 0.020(ad)	0.161 ± 0.023(ad)	0.147 ± 0.017(cd)
**Bacilli**	0.058 ± 0.010(a)	0.067 ± 0.010(a)	0.133 ± 0.024(b)	0.059 ± 0.013(a)	0.212 ± 0.037(c)	0.100 ± 0.023(d)	0.095 ± 0.015(de)	0.056 ± 0.021(a)	0.084 ± 0.017(e)
**Bacteroidia**	0.012 ± 0.004(de)	0.018 ± 0.005(a)	0.040 ± 0.012(b)	0.033 ± 0.007(c)	0.009 ± 0.004(d)	0.015 ± 0.005(ae)	0.013 ± 0.004(de)	0.013 ± 0.005(de)	0.012 ± 0.004(de)
**Blastocatellia**	0.023 ± 0.005(g)	0.012 ± 0.004(a)	0.005 ± 0.002(b)	0.008 ± 0.004(bc)	0.001 ± 0.001(d)	0.018 ± 0.005(e)	0.010 ± 0.003(ac)	0.029 ± 0.009(f)	0.013 ± 0.004(a)
**Chloroflexia**	0.020 ± 0.007(e)	0.010 ± 0.004(ab)	0.009 ± 0.003(a)	0.014 ± 0.005(bc)	0.004 ± 0.002(d)	0.011 ± 0.005(ab)	0.011 ± 0.003(ab)	0.015 ± 0.006(c)	0.012 ± 0.003(ab)

**Clostridia**	0.016 ± 0.004$^{(b)}$	0.019 ± 0.007$^{(ab)}$	0.007 ± 0.003$^{(c)}$	0.028 ± 0.007$^{(d)}$	0.006 ± 0.003$^{(c)}$	0.023 ± 0.006$^{(e)}$	0.021 ± 0.005$^{(ae)}$	0.011 ± 0.007$^{(f)}$	0.023 ± 0.005$^{(e)}$
**Gammaproteobacteria**	0.060 ± 0.008$^{(b)}$	0.070 ± 0.008$^{(a)}$	0.056 ± 0.010$^{(b)}$	0.074 ± 0.009$^{(a)}$	0.042 ± 0.008$^{(c)}$	0.049 ± 0.008$^{(d)}$	0.057 ± 0.006$^{(b)}$	0.055 ± 0.010$^{(bd)}$	0.055 ± 0.007$^{(bd)}$
**Gemmatimonadetes**	0.026 ± 0.006$^{(c)}$	0.016 ± 0.005$^{(a)}$	0.016 ± 0.005$^{(a)}$	0.015 ± 0.005$^{(a)}$	0.016 ± 0.004$^{(a)}$	0.017 ± 0.005$^{(a)}$	0.021 ± 0.005$^{(b)}$	0.030 ± 0.007$^{(c)}$	0.021 ± 0.006$^{(b)}$
**KD4-96**	0.032 ± 0.006$^{(a)}$	0.029 ± 0.006$^{(abc)}$	0.014 ± 0.005$^{(d)}$	0.031 ± 0.007$^{(ab)}$	0.009 ± 0.005$^{(e)}$	0.025 ± 0.006$^{(f)}$	0.026 ± 0.005$^{(cf)}$	0.027 ± 0.004$^{(bcf)}$	0.032 ± 0.005$^{(a)}$
**MB-A2-108**	0.014 ± 0.003$^{(de)}$	0.019 ± 0.004$^{(ab)}$	0.002 ± 0.001$^{(c)}$	0.013 ± 0.004$^{(d)}$	0.004 ± 0.002$^{(c)}$	0.021 ± 0.005$^{(a)}$	0.015 ± 0.003$^{(de)}$	0.016 ± 0.004$^{(be)}$	0.019 ± 0.004$^{(ab)}$
**Nitrospiria**	0.007 ± 0.003$^{(ce)}$	0.009 ± 0.002$^{(abc)}$	0.004 ± 0.002$^{(d)}$	0.011 ± 0.004$^{(b)}$	0.003 ± 0.002$^{(d)}$	0.007 ± 0.002$^{(e)}$	0.009 ± 0.002$^{(ac)}$	0.009 ± 0.004$^{(abc)}$	0.009 ± 0.003$^{(ab)}$
**Phycisphaerae**	0.013 ± 0.005$^{(c)}$	0.007 ± 0.003$^{(ab)}$	0.010 ± 0.004$^{(a)}$	0.006 ± 0.002$^{(b)}$	0.015 ± 0.005$^{(c)}$	0.007 ± 0.003$^{(ab)}$	0.013 ± 0.004$^{(c)}$	0.010 ± 0.004$^{(a)}$	0.008 ± 0.002$^{(ab)}$
**Planctomycetes**	0.025 ± 0.007$^{(ac)}$	0.023 ± 0.006$^{(abc)}$	0.031 ± 0.008$^{(d)}$	0.028 ± 0.005$^{(ad)}$	0.019 ± 0.007$^{(b)}$	0.026 ± 0.007$^{(acd)}$	0.026 ± 0.006$^{(acd)}$	0.019 ± 0.006$^{(b)}$	0.022 ± 0.007$^{(bc)}$
**Polyangia**	0.010 ± 0.003$^{(ad)}$	0.013 ± 0.004$^{(a)}$	0.025 ± 0.007$^{(b)}$	0.018 ± 0.004$^{(c)}$	0.009 ± 0.006$^{(d)}$	0.012 ± 0.003$^{(ad)}$	0.012 ± 0.003$^{(ad)}$	0.012 ± 0.005$^{(ad)}$	0.010 ± 0.003$^{(ad)}$
**Thermoleophilia**	0.079 ± 0.015$^{(a)}$	0.077 ± 0.011$^{(a)}$	0.093 ± 0.018$^{(bc)}$	0.059 ± 0.011$^{(d)}$	0.095 ± 0.017$^{(b)}$	0.083 ± 0.012$^{(ac)}$	0.078 ± 0.012$^{(a)}$	0.081 ± 0.013$^{(a)}$	0.100 ± 0.013$^{(b)}$
**Verrucomicrobiae**	0.099 ± 0.022$^{(bf)}$	0.089 ± 0.023$^{(ab)}$	0.147 ± 0.026$^{(c)}$	0.063 ± 0.019$^{(d)}$	0.121 ± 0.034$^{(e)}$	0.080 ± 0.022$^{(ad)}$	0.109 ± 0.020$^{(ef)}$	0.100 ± 0.026$^{(bf)}$	0.109 ± 0.021$^{(ef)}$
**Vicinamibacteria**	0.081 ± 0.013$^{(cf)}$	0.112 ± 0.018$^{(ab)}$	0.074 ± 0.014$^{(c)}$	0.117 ± 0.022$^{(a)}$	0.009 ± 0.008$^{(d)}$	0.100 ± 0.021$^{(be)}$	0.081 ± 0.018$^{(cf)}$	0.069 ± 0.017$^{(c)}$	0.092 ± 0.018$^{(ef)}$

Table S2: Relative abundance of classes. Values are presented as mean ± standard deviation (SD) (with Tukey group letters).

Table S3: Frequency of Culture Independent Bacterial Classes per Soil type

Class	**Clay loam**	**Clay**	**Silt clay loam**	**Sandy loam**
**Acidimicrobiia**	0.035 ± 0.006$^{(a)}$	0.036 ± 0.015$^{(a)}$	0.024 ± 0.012$^{(b)}$	0.032 ± 0.008$^{(a)}$
**Acidobacteriae**	0.034 ± 0.018$^{(ac)}$	0.025 ± 0.016$^{(a)}$	0.055 ± 0.038$^{(b)}$	0.044 ± 0.023$^{(c)}$
**Actinobacteria**	0.114 ± 0.020$^{(b)}$	0.128 ± 0.024$^{(a)}$	0.114 ± 0.028$^{(b)}$	0.116 ± 0.027$^{(b)}$
**Alphaproteobacteria**	0.159 ± 0.026$^{(b)}$	0.130 ± 0.023$^{(a)}$	0.156 ± 0.026$^{(b)}$	0.155 ± 0.021$^{(b)}$
**Bacilli**	0.062 ± 0.011$^{(d)}$	0.097 ± 0.042$^{(a)}$	0.156 ± 0.064$^{(b)}$	0.078 ± 0.024$^{(c)}$
**Bacteroidia**	0.015 ± 0.005$^{(b)}$	0.037 ± 0.010$^{(a)}$	0.012 ± 0.005$^{(b)}$	0.013 ± 0.004$^{(b)}$
**Blastocatellia**	0.018 ± 0.007$^{(b)}$	0.006 ± 0.003$^{(a)}$	0.009 ± 0.009$^{(a)}$	0.017 ± 0.010$^{(b)}$

**Chloroflexia**	0.015 ± 0.007(c)	0.011 ± 0.005(a)	0.007 ± 0.005(b)	0.013 ± 0.004(a)
**Clostridia**	0.017 ± 0.006(ab)	0.017 ± 0.012(ab)	0.015 ± 0.010(a)	0.019 ± 0.008(b)
**Gammaproteobacteria**	0.065 ± 0.009(a)	0.065 ± 0.013(a)	0.046 ± 0.008(b)	0.056 ± 0.008(c)
**Gemmatimonadetes**	0.021 ± 0.007(c)	0.015 ± 0.005(a)	0.017 ± 0.004(a)	0.024 ± 0.007(b)
**KD4-96**	0.030 ± 0.006(c)	0.022 ± 0.010(a)	0.017 ± 0.010(b)	0.028 ± 0.006(c)
**MB-A2-108**	0.016 ± 0.004(c)	0.007 ± 0.006(a)	0.012 ± 0.009(b)	0.017 ± 0.004(c)
**Nitrospiria**	0.008 ± 0.002(ac)	0.008 ± 0.004(a)	0.005 ± 0.003(b)	0.009 ± 0.003(c)
**Phycisphaerae**	0.010 ± 0.005(ab)	0.008 ± 0.003(a)	0.011 ± 0.006(b)	0.010 ± 0.004(b)
**Planctomycetes**	0.024 ± 0.007(b)	0.029 ± 0.007(a)	0.022 ± 0.008(b)	0.023 ± 0.007(b)
**Polyangia**	0.012 ± 0.004(b)	0.022 ± 0.007(a)	0.011 ± 0.005(b)	0.011 ± 0.004(b)
**Thermoleophilia**	0.078 ± 0.013(a)	0.076 ± 0.023(a)	0.089 ± 0.016(b)	0.086 ± 0.016(b)
**Verrucomicrobiae**	0.094 ± 0.023(a)	0.106 ± 0.048(a)	0.100 ± 0.035(a)	0.106 ± 0.023(a)
**Vicinamibacteria**	0.096 ± 0.022(a)	0.095 ± 0.028(a)	0.055 ± 0.048(b)	0.080 ± 0.020(c)

Table S3: Relative abundance of classes. Values are presented as mean ± standard deviation (SD) (with Tukey group letters).

Culture independent bacterial community phylogenetic β-diversity

To assess differences in community structure across samples, we performed non-metric multidimensional scaling based on weighted-Unifrac distance (NMDS; Figs. S10A–C). Among the tested factors, location exerted the strongest influence on community variation (ANOSIM R = 0.8814, p = 0.001; PERMANOVA F = 38.3068, R^2 = 0.5062, p = 0.001; Figs. S10B, G). Soil type also contributed substantially, though to a lesser extent (ANOSIM R = 0.4419, p = 0.001; PERMANOVA F = 22.4523, R^2 = 0.1814, p = 0.001; Figs. S10C, G). In contrast, crop identity had only a minor effect on community composition (ANOSIM R = 0.0269, p = 0.001; PERMANOVA F = 1.800, R^2 = 0.0346, p = 0.001; Figs. S10A, G). Overall, both ANOSIM and PERMANOVA consistently indicated location as the dominant driver of community variation, followed by soil type, with crop showing the weakest influence (Fig. S10G).

Additionally, to evaluate the community composition changes considering the compositional nature of the sequencing data, the ASV table was normalised using centred log-ratio (clr). The resulting table was used for principal component analysis (PCA) followed by Universal Manifold Approximation and Projection for Dimension Reduction (UMAP) (Figs. S11-A-C) and ANOSIM and PERMANOVA tests using Aitchison distance. As observed for the analyses using Bray-Curtis and w-Unifrac, location exerted the strongest influence on community variation (ANOSIM R = 0.931, p = 0.001; PERMANOVA F = 19.8168, R^2 = 0.3465, p = 0.001; Figs. S11B, G). Soil has shown a smaller effect (ANOSIM R = 0.428, p = 0.001; PERMANOVA F = 15.8311, R^2 = 0.1315, p = 0.001; Figs. S11C, G). While crop identity had the lowest effect on community composition (ANOSIM R = 0.047, p = 0.001; PERMANOVA F = 2.0713, R^2 = 0.0397, p = 0.001; Figs. S11A, G). Overall, both ANOSIM and PERMANOVA consistently indicated location as the dominant driver of community variation, followed by soil type, with crop showing the weakest influence (Fig. S11G).

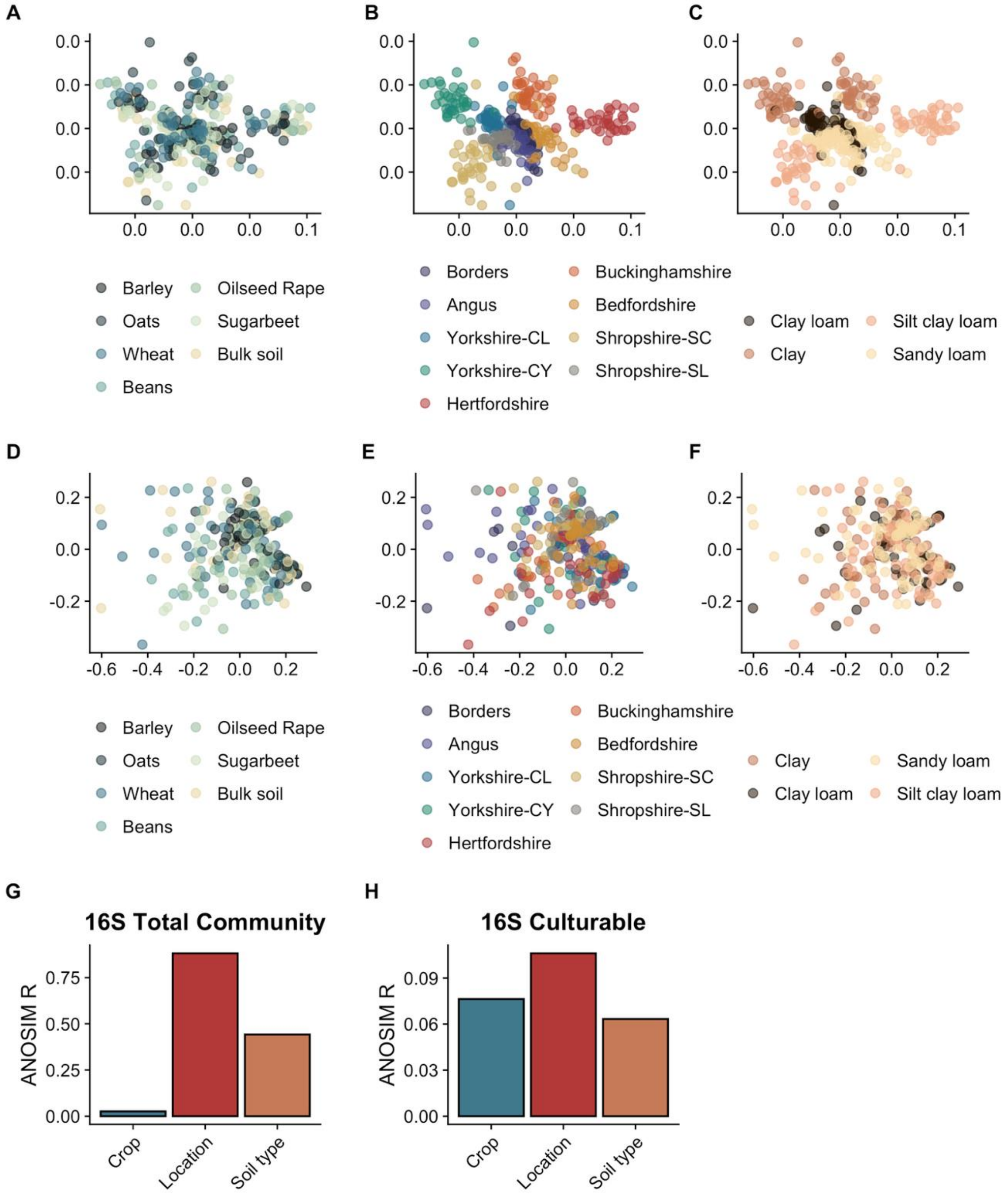

Figure S10: NMDS ordination of bacterial communities based on 16S rRNA gene ASVs and culture functional profiles and ANOSIM R values. Non-metric multidimensional scaling (NMDS) plots based on weighted-Unifrac distance. NMDS1 and NMDS2 are represented in the x and y axis, respectively. Panels A-C show the culture independent bacterial community profiles (stress value of 0.1405), D-F the cultured bacterial profiles (stress value of 0.1996). Panel (A, D) shows samples grouped and coloured by crop type, (B, E) by location, and (C, F) by soil type. Each point represents a sample, and colours correspond to the grouping variable shown in each panel, revealing patterns in community structure across environmental categories. Panels G-H show ANOSIM R values based on the data shown in plots A-F respectively.

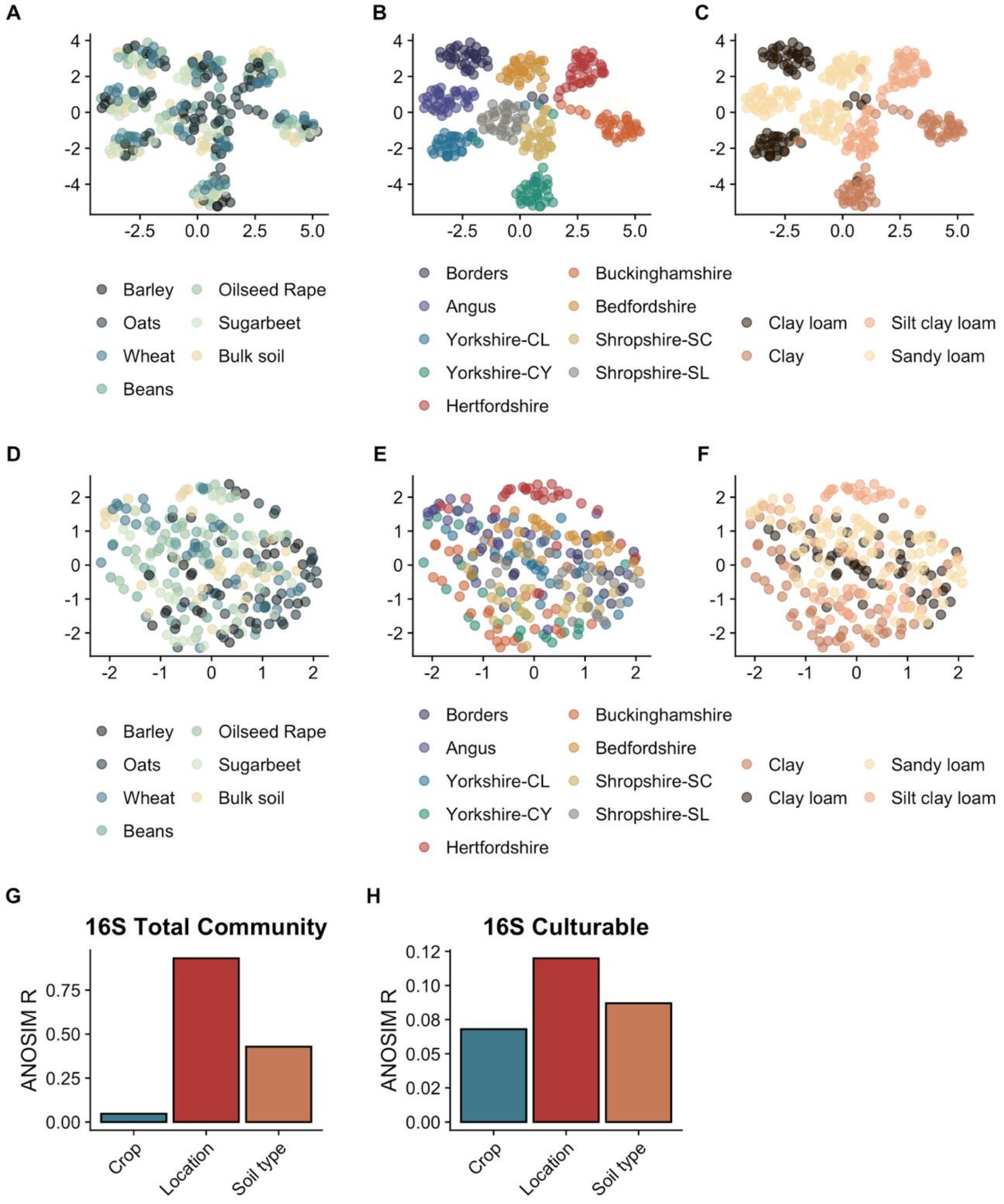

Figure S11: Universal Manifold Approximation and Projection for Dimension Reduction (UMAP) ordination of bacterial communities based on 16S rRNA gene ASVs and culture functional profiles and ANOSIM R values. Amplicon sequence variant (ASV) tables were transformed using centred log-ratio (CLR) normalization and reduced using principal component analysis (50 PCs). The resulting coordinates were embedded using UMAP (100 neighbours, min_dist = 0.5). Panels A-C show the culture independent bacterial community profiles, D-F the cultured bacterial profiles. Panel (A, D) shows samples grouped and coloured by crop type, (B, E) by location, and (C, F) by soil type. Each point represents a sample, and colours correspond to the grouping variable shown in each panel, revealing patterns in community structure across environmental categories. Panels G-H show ANOSIM R values based on the data shown in plots A-F respectively.

Cultivated bacterial community phylogenetic α-diversity

The normalised ASV table served as the basis for calculating Faith's alpha-diversity metrics (Figs. S7D–F). Diversity indices varied significantly only according to crop ($p < 0.001$). Across crops, oats and barley samples displayed significantly reduced diversity to the other crop types ($n = 45$) (Fig. S7D). Among locations ($n = 35$) (Fig. S7E) and soil type (Fig. S7F) ANOVA indicated that these treatments did not affect this diversity index significantly. The DivNet estimation of H' (Fig. S8D-F) shown a similar profile of diversity as was observed for the other diversity indexes. Also, it indicated that the factors analysed had a significant effect on the community based on the betta test ($p < 0.0001$).

Cultivated bacterial taxonomy

The taxonomic classification of the ASVs indicated a dominance of five classes: Actinobacteria, Alphaproteobacteria, Bacilli, Bacteroidia, and Gammaproteobacteria across all samples (Fig. S12). In all treatments, Actinobacteria was the most common class, followed by either Bacilli or Gammaproteobacteria. Variations in the Class frequencies between locations (Fig. S12B, Table S5) are greater than between crops (Fig. S12A, Table S4) and soil types (Fig. S12C, Table S6). Actinobacteria variation was 27-60%, 45-60%, 37-55%, between locations, crops, and soil types, respectively. This pattern was similar for the other dominant classes.

The order Micrococcales was the most common culture in all treatments (Fig. S13). Xanthomonadales were high in Yorkshire-CL and Hertfordshire, while Pseudomonadales was most abundant in Angus. Additionally, Rhizobiales were more frequent in Hertfordshire and Streptomycetales in Yorkshire-CY and Buckinghamshire. Conversely, Burkholderia were markedly low in Angus.

Rhizobiales were present in high relative abundance in beans. Additionally, Sphingomonadales were more frequent in sugar beet. Propionibacteriales (Nocardia) and Burkholderia were lower in oats compared to other crops. Similarly, Streptomycetales were low in bulk soil samples, but high in oats and clay.

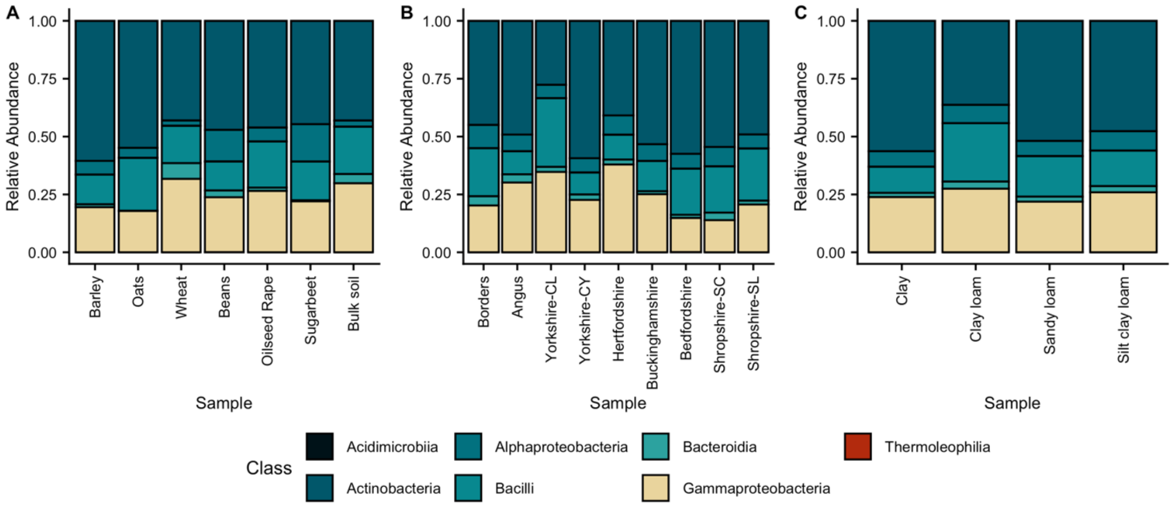

Figure S12: Taxonomic composition of cultured bacterial ASVs across samples grouped by environmental variables. Stacked bar charts showing the relative abundance of cultured bacterial amplicon sequence variants (ASVs) derived from 16S rRNA gene sequencing, assigned at the taxonomic level of class. Bars are coloured according to taxonomic class affiliation. Panel (A) shows

samples grouped by crop type, (B) by location, and (C) by soil type. Each bar represents the composite microbial community profile for the respective grouping.

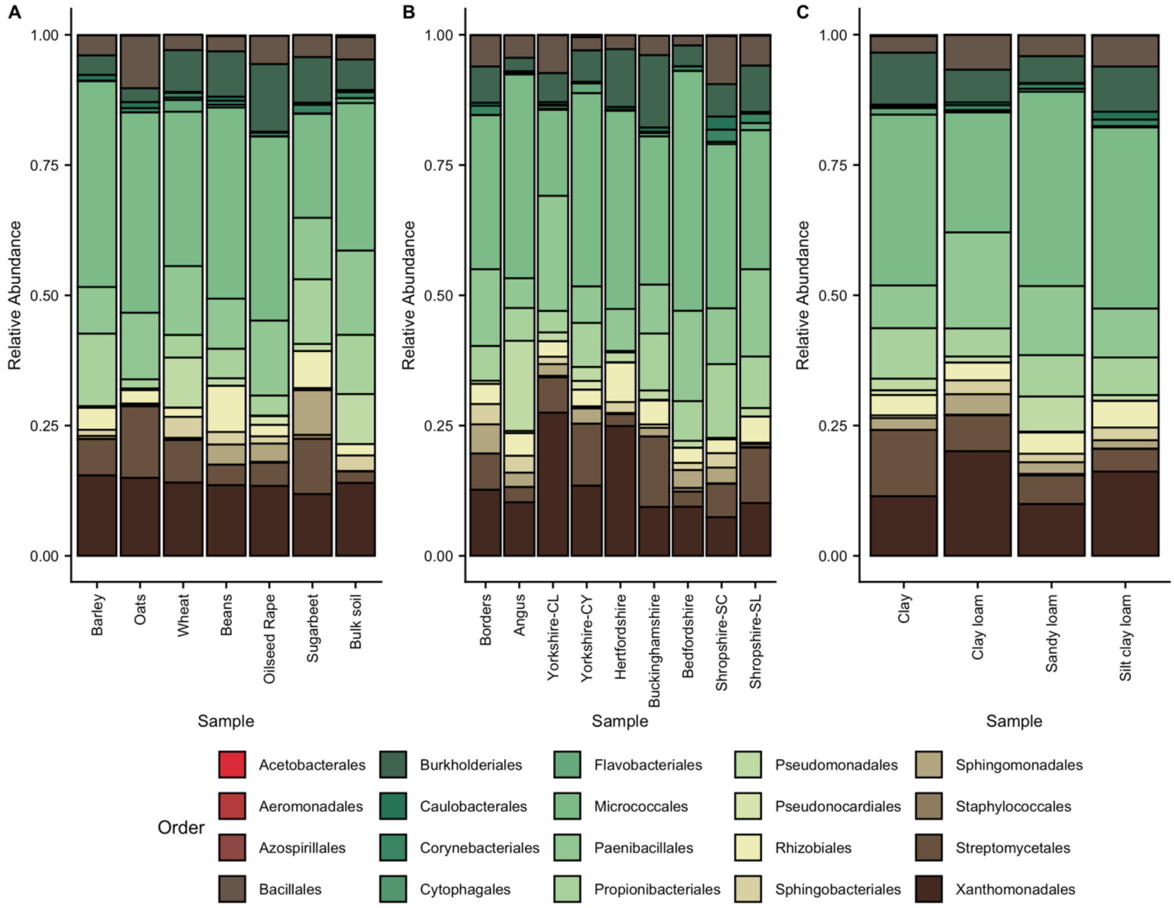

Figure S13: Taxonomic composition of cultured bacterial ASVs across samples grouped by environmental variables. Stacked bar charts showing the relative abundance of uncultured bacterial amplicon sequence variants (ASVs) derived from 16S rRNA gene sequencing, assigned at the taxonomic level of order. Bars are coloured according to taxonomic class affiliation. Panel (A) shows samples grouped by crop type, (B) by location, and (C) by soil type. Each bar represents the composite microbial community profile for the respective grouping, highlighting variation in the class-level composition of cultured bacteria across environmental contexts.

Table S4: Frequency of Cultured Bacterial Classes per Crop.

Class	Barley	Beans	Bulk soil	Oats	Oilseed rape	Sugarbeet	Wheat
**Acidimicrobiia**	0.000 ± 0.000(a)	0.000 ± 0.000(a)	0.000 ± 0.000(a)	0.000 ± 0.000(a)	0.000 ± 0.000(a)	0.000 ± 0.000(a)	0.000 ± 0.000(a)
**Actinobacteria**	0.605 ± 0.228(b)	0.471 ± 0.247(ab)	0.431 ± 0.222(a)	0.549 ± 0.230(ab)	0.461 ± 0.248(ab)	0.446 ± 0.233(ab)	0.431 ± 0.223(a)
**Alphaproteobacteria**	0.059 ± 0.076(c)	0.136 ± 0.190(ab)	0.026 ± 0.050(c)	0.043 ± 0.089(c)	0.060 ± 0.098(ac)	0.161 ± 0.138(b)	0.023 ± 0.061(c)
**Anaerolineae**	0.000 ± 0.000(a)	0.000 ± 0.000(a)	0.000 ± 0.000(a)	0.000 ± 0.000(a)	0.000 ± 0.000(a)	0.000 ± 0.000(a)	0.000 ± 0.000(a)
**Bacilli**	0.129 ± 0.117(a)	0.125 ± 0.230(a)	0.205 ± 0.186(a)	0.229 ± 0.168(a)	0.200 ± 0.228(a)	0.168 ± 0.195(a)	0.162 ± 0.182(a)
**Bacteroidia**	0.012 ± 0.047(ab)	0.029 ± 0.071(ab)	0.040 ± 0.140(ab)	0.001 ± 0.005(a)	0.014 ± 0.064(ab)	0.004 ± 0.023(a)	0.068 ± 0.114(b)
**Gammaproteobacteria**	0.195 ± 0.238(a)	0.237 ± 0.192(a)	0.299 ± 0.252(a)	0.179 ± 0.212(a)	0.266 ± 0.218(a)	0.220 ± 0.182(a)	0.318 ± 0.252(a)
**Thermoleophilia**	0.000 ± 0.000(a)	0.001 ± 0.003(a)	0.000 ± 0.000(a)	0.000 ± 0.000(a)	0.000 ± 0.000(a)	0.000 ± 0.000(a)	0.000 ± 0.000(a)

Mean ± SD (with Tukey group letters) of Class abundance per Crop.

Table S5: Frequency of Cultured Bacterial Classes per Location.

Class	Borders	Yorkshire-CL	Buckinghamshire	Yorkshire-CY	Hertfordshire	Shropshire-SC	Angus	Bedfordshire	Shropshire-SL
**Acidimicrobiia**	0.000 ± 0.000(a)	0.000 ± 0.000(a)	0.000 ± 0.000(a)	0.000 ± 0.000(a)	0.000 ± 0.000(a)	0.000 ± 0.000(a)	0.000 ± 0.000(a)	0.000 ± 0.000(a)	0.000 ± 0.000(a)
**Actinobacteria**	0.450 ± 0.252(ab)	0.276 ± 0.206(a)	0.533 ± 0.197(b)	0.593 ± 0.210(b)	0.409 ± 0.211(ab)	0.545 ± 0.238(b)	0.491 ± 0.238(b)	0.575 ± 0.245(b)	0.491 ± 0.207(b)
**Alphaproteobacteria**	0.100 ± 0.174(a)	0.058 ± 0.090(a)	0.072 ± 0.131(a)	0.061 ± 0.101(a)	0.083 ± 0.135(a)	0.084 ± 0.130(a)	0.072 ± 0.112(a)	0.064 ± 0.089(a)	0.060 ± 0.100(a)
**Anaerolineae**	0.000 ± 0.000(a)	0.000 ± 0.000(a)	0.000 ± 0.000(a)	0.000 ± 0.000(a)	0.000 ± 0.000(a)	0.000 ± 0.000(a)	0.000 ± 0.000(a)	0.000 ± 0.000(a)	0.000 ± 0.000(a)
**Bacilli**	0.208 ± 0.207(ab)	0.297 ± 0.291(a)	0.131 ± 0.119(b)	0.095 ± 0.108(b)	0.107 ± 0.169(b)	0.200 ± 0.208(ab)	0.100 ± 0.119(b)	0.199 ± 0.163(ab)	0.226 ± 0.185(ab)
**Bacteroidia**	0.040 ± 0.144(a)	0.022 ± 0.072(a)	0.013 ± 0.041(a)	0.023 ± 0.069(a)	0.021 ± 0.061(a)	0.032 ± 0.094(a)	0.035 ± 0.082(a)	0.014 ± 0.066(a)	0.016 ± 0.072(a)
**Gammaproteobacteria**	0.203 ± 0.169(ab)	0.347 ± 0.261(a)	0.251 ± 0.189(ab)	0.227 ± 0.209(ab)	0.380 ± 0.223(a)	0.139 ± 0.193(b)	0.302 ± 0.279(ab)	0.148 ± 0.165(b)	0.207 ± 0.212(ab)
**Thermoleophilia**	0.000 ± 0.000(a)	0.000 ± 0.000(a)	0.000 ± 0.000(a)	0.000 ± 0.000(a)	0.000 ± 0.000(a)	0.000 ± 0.000(a)	0.000 ± 0.000(a)	0.001 ± 0.004(a)	0.000 ± 0.000(a)

Mean ± SD (with Tukey group letters) of Class abundance per Soil type.

Table S6: Frequency of Cultured Bacterial Classes per Soil Type.

Class	Clay	Clay loam	Sandy loam	Silt clay loam
**Acidimicrobiia**	0.000 ± 0.000$^{(a)}$	0.000 ± 0.000$^{(a)}$	0.000 ± 0.000$^{(a)}$	0.000 ± 0.000$^{(a)}$
**Actinobacteria**	0.563 ± 0.204$^{(b)}$	0.363 ± 0.244$^{(a)}$	0.519 ± 0.231$^{(b)}$	0.477 ± 0.233$^{(b)}$
**Alphaproteobacteria**	0.067 ± 0.116$^{(a)}$	0.079 ± 0.139$^{(a)}$	0.065 ± 0.100$^{(a)}$	0.084 ± 0.132$^{(a)}$
**Anaerolineae**	0.000 ± 0.000$^{(a)}$	0.000 ± 0.000$^{(a)}$	0.000 ± 0.000$^{(a)}$	0.000 ± 0.000$^{(a)}$
**Bacilli**	0.113 ± 0.114$^{(b)}$	0.252 ± 0.254$^{(a)}$	0.175 ± 0.166$^{(ab)}$	0.154 ± 0.193$^{(b)}$
**Bacteroidia**	0.018 ± 0.056$^{(a)}$	0.031 ± 0.113$^{(a)}$	0.022 ± 0.074$^{(a)}$	0.027 ± 0.079$^{(a)}$
**Gammaproteobacteria**	0.239 ± 0.198$^{(a)}$	0.275 ± 0.230$^{(a)}$	0.219 ± 0.230$^{(a)}$	0.259 ± 0.240$^{(a)}$
**Thermoleophilia**	0.000 ± 0.000$^{(a)}$	0.000 ± 0.000$^{(a)}$	0.000 ± 0.002$^{(a)}$	0.000 ± 0.000$^{(a)}$

Mean ± SD (with Tukey group letters) of Class abundance per Location.

Cultured bacterial community phylogenetic β-diversity

To assess differences in community structure across samples, we performed non-metric multidimensional scaling based on weighted-Unifrac distance (NMDS; Figs. S10D-F). Among the tested factors, location exerted the strongest influence on community variation (ANOSIM R = 0.1061, p = 0.001; PERMANOVA F = 3.7716, R^2 = 0.1105, p = 0.001; Figs. S10E, H). Soil type also contributed substantially, though to a lesser extent (ANOSIM R = 0.0633, p = 0.001; PERMANOVA F = 3.832, R^2 = 0.0443, p = 0.001; Figs. S10F, H). In contrast to what was observed on the uncultured bacterial community, crop identity had a greater effect than soil type community composition, although it was only minor (ANOSIM R = 0.0763, p = 0.001; PERMANOVA F = 3.5755, R^2 = 0.0805, p = 0.001; Figs. S10D, H). Overall, both ANOSIM and PERMANOVA consistently indicated location as the dominant driver of community variation, followed by crop, with soil type showing the weakest influence (Fig. S10H). We can also observe that the effects observed using w-Unifrac were smaller than those using Bray-Curtis distances. These differences can be explained by the stronger selection of plating of certain phylogenetic groups. Although we can observe changes in the ASVs present they are from limited phylogenetic diversity (Fig. S10).

The UMAP (Figs. S11D-F), ANOSIM and PERMANOVA tests using Aitchison distance shown a similar trend as observed above. As observed before, location exerted the strongest influence on community variation (ANOSIM R = 0.120, p = 0.001; PERMANOVA F = 2.3284, R^2 = 0.0712, p = 0.001; Figs. S11E, H). Soil has shown a smaller effect (ANOSIM R = 0.087, p = 0.001; PERMANOVA F = 2.6071, R^2 = 0.0306, p = 0.001; Figs. S11F, H). While crop identity had the lowest effect on community composition (ANOSIM R = 0.068, p = 0.001; PERMANOVA F = 2.0118, R^2 = 0.047, p = 0.001; Figs. S11D, H). Overall, both ANOSIM and PERMANOVA indicated location as the dominant driver of community variation, followed by soil type, with crop showing the weakest influence (Fig. S11G).

Supplementary references

Aleksandrov, V.G., Blagodyr, R.N., Il'ev, I.P., 1967. [Phosphorus acid isolation from apatite produced by silicate bacteria]. Mikrobiol Zh 29, 111-114.

Frazier, W.C., Rupp, P., 1928. Studies on the Proteolytic Bacteria of Milk Iii. Action of Proteolytic Bacteria of Milk on Casein and Gelatin. J Bacteriol 16, 187-196

Louden, B.C., Haarmann, D., Lynne, A.M., 2011. Use of Blue Agar CAS Assay for Siderophore Detection. J Microbiol Biol Educ 12, 51-53.

Penrose, D.M., Glick, B.R., 2003. Methods for isolating and characterizing ACC deaminase-containing plant growth-promoting rhizobacteria. Physiol Plant 118, 10-15.

Pikovskaya, R.I., 1948. Mobilization of phosphorus in soil in connection with the vital activity of some microbial species. Mikrobiologiya 17, 362–370.

Schwyn, B., Neilands, J.B., 1987. Universal chemical assay for the detection and determination of siderophores. Anal Biochem 160, 47-56.

Subba Rao, N.S., 1977. Soil Microorganisms and Plant Growth. Oxford & IBH Pub. Co., New Delhi, 289 p.
